# Synergistic Effect of Plasmonic Gold Nanoparticles Decorated Carbon Nanotubes in Quantum Dots/TiO_2_ for Optoelectronic Devices

**DOI:** 10.1002/advs.202001864

**Published:** 2020-08-26

**Authors:** Gurpreet Singh Selopal, Mahyar Mohammadnezhad, Lucas V. Besteiro, Ozge Cavuslar, Jiabin Liu, Hui Zhang, Fabiola Navarro‐Pardo, Guiju Liu, Maorong Wang, Emek G. Durmusoglu, Havva Yagci Acar, Shuhui Sun, Haiguang Zhao, Zhiming M. Wang, Federico Rosei

**Affiliations:** ^1^ Institute of Fundamental and Frontier Sciences University of Electronic Science and Technology of China Chengdu 610054 P. R. China; ^2^ Centre Énergie Matériaux et Télécommunications Institut National de la Recherché Scientifique 1650 Boul. Lionel Boulet Varennes Québec J3X 1S2 Canada; ^3^ Department of Chemistry Koc University Rumelifeneri Yolu, Sariyer Istanbul 34450 Turkey; ^4^ State Key Laboratory of Bio‐Fibers and Eco‐Textiles & College of Physics Qingdao University No. 308 Ningxia Road Qingdao 266071 P. R. China

**Keywords:** Au:carbon nanotubes hybrid networks, carbon nanotubes, gold nanoparticles, photoelectrochemical cells, plasmonic nanoparticles

## Abstract

Here, a facile approach to enhance the performance of solar‐driven photoelectrochemical (PEC) water splitting is described by means of the synergistic effects of a hybrid network of plasmonic Au nanoparticles (NPs) decorated on multiwalled carbon nanotubes (CNTs). The device based on TiO_2_–Au:CNTs hybrid network sensitized with colloidal CdSe/(CdSe*_x_*S_1−_ *_x_*)_5_/(CdS)_1_ core/alloyed shell quantum dots (QDs) yields a saturated photocurrent density of 16.10 ± 0.10 mA cm^−2^ [at 1.0 V vs reversible hydrogen electrode (RHE)] under 1 sun illumination (AM 1.5G, 100 mW cm^−2^), which is ≈26% higher than the control device. The in‐depth mechanism behind this significant improvement is revealed through a combined experimental and theoretical analysis for QDs/TiO_2_–Au:CNTs hybrid network and demonstrates the multifaceted impact of plasmonic Au NPs and CNTs: i) hot‐electron injection from Au NPs into CNTs and TiO_2_; ii) near‐field enhancement of the QDs absorption and carrier generation/separation processes by the plasmonic Au NPs; iii) enhanced photoinjected electron transport due to the highly directional pathways offered by CNTs. These results provide fundamental insights on the properties of QDs/TiO_2_–Au:CNTs hybrid network, and highlights the possibility to improve the performance of other solar technologies.

## Graphical abstract

The performance of solar‐driven photoelectrochemical (PEC) devices is enhanced through the synergistic effects of plasmonic Au nanoparticles and multiwalled carbon nanotubes (CNTs). A PEC device based on alloyed core/shell quantum dot sensitized TiO_2_–Au:CNTs hybrid photoanode yields a saturated photocurrent density of 16.10 ± 0.10 mA cm^−2^ [1.0 V vs reversible hydrogen electrode (RHE)]. This approach offers a promising solution to improve the performance of optoelectronic devices.

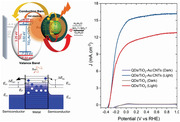

## 1 Introduction

Solar‐driven hydrogen (H_2_) generation is a promising approach for the direct conversion of solar energy into a clean and renewable fuel, to partially address the future energy demands and related environmental issues.^[^ ^1^ ^]^ A typical solar‐driven water splitting system exploits two‐half chemical reactions such as the reduction of water to H_2_ at the cathode and oxidation of water to oxygen (O_2_) at the anode. For this purpose, the starting point is to optimize the band alignment of a semiconductor photoanode and photocathode with suitable reduction and oxidation potential of the electrolyte. A photon absorbed by the semiconductor photoanode with energy higher than its bandgap generates an electron–hole pair (exciton), which is dissociated at the semiconductor–electrolyte interface and leads to hole oxidation and electron transfer through an external circuit to the cathode for H_2_ generation.

Following the pioneering work by Fujishima and Honda,^[^ ^2^ ^]^ several semiconductors have been studied for hydrogen generation, including TiO_2_,^[^ ^3^ ^]^ ZnO,^[^ ^4^ ^]^ SnO_2_,^[^ ^5^ ^]^ Cu_2_O,^[^ ^6^ ^]^ WO_3_,^[^ ^7^ ^]^ and Fe_2_O_3_.^[^ ^8^ ^]^ In particular, TiO_2_ presents several advantages, as it is a low cost, nontoxic, efficient, and stable.^[^ ^9^ ^]^ However, due to its wide bandgap (3.2 eV), the absorption of TiO_2_ is limited to the ultraviolet (UV) spectral region (which represents only a 3–5% of the full solar spectrum).^[^ ^10^ ^]^ In addition, the low electron mobility (1 cm^2^ V^−1^ s^−1^) and short carrier diffusion length (10–100 nm) of TiO_2_ result in an overall low efficiency of H_2_ generation.^[^ ^11^ ^]^

Multidisciplinary approaches have been explored to address these challenges. For example, sensitizing the TiO_2_ anode with colloidal quantum dots (QDs) is a promising approach to extend the absorption spectrum toward the visible and near‐infrared (NIR) region.^[^ ^12^ ^]^ The properties of QDs such as size/shape/composition‐tunable absorption edge,^[^ ^13^ ^]^ their high absorption coefficient^[^ ^14^ ^]^ and the possibility of multiple exciton generation (MEG)^[^ ^15^ ^]^ by single photon absorption allow to significantly enhance the performance of photoelectrochemical (PEC) cells for H_2_ generation. Similarly, the efficient transport of photogenerated electrons from the QDs to the collecting electrode surface through the network of wide bandgap semiconductor mesoporous film without quenching at grain boundaries is another key aspect. In this context, the design and synthesis of one‐dimensional (1D) (e.g., nanowires,^[^ ^16^ ^]^ nanorods,^[^ ^17^ ^]^ and nanotubes^[^ ^18^ ^]^), branched nanorods and hierarchical nanotubes^[^ ^19^ ^]^ of TiO_2_ have been extensively studied to further improve performance.

Recently, it has been shown that combining localized surface plasmon resonance (LSPR) of noble metal (e.g., Au or Ag) nanoparticles (NPs) with wide‐bandgap semiconductors (e.g., TiO_2_) is a promising approach to enhance the light harvesting efficiency of solar technologies.^[^ ^20^ ^]^ These plasmonic NPs work as nanoantennas that localize the impinging light's energy, to then transfer a fraction of it to its environment through different optical and charge‐transfer mechanisms.^[^ ^21^ ^]^ However, plasmonic effects only originate from nanoscale noble metal crystals, and their proximity to other systems also causes charge trapping, thereby hampers device performance.^[^ ^22^ ^]^

To address this issue, we propose to use 1D materials capable of promoting the efficient separation and transport of photogenerated charge carriers.^[^ ^23^ ^]^ In this context, carbon nanomaterials such as multiwall carbon nanotubes (CNTs), graphene, graphene nanoribbons, and graphene oxide may be useful for enhancing charge carrier transport and collection.^[^ ^24^ ^]^ In particular, CNTs could be the most suitable candidates due to their unique transport and structural properties.^[^ ^25^ ^]^ The addition of a precise amount of CNTs in TiO_2_ mesoporous paste boosts the performance of solar technologies thanks to improved electron transport and reduced carrier recombination within the photoanode.^[^ ^24^, ^26^ ^]^ Recently, Lee et al. demonstrated that plasmonic metal NPs–CNTs hybrid networks lead to significant suppression of charge trapping and related nonradiative recombination that are typical of plasmonic NPs.^[^ ^22a^ ^]^ To the best of our knowledge, there is no report exploring the use of plasmonic metal NPs–CNTs hybrid networks in colloidal QD based PEC devices for H_2_ production. In particular, the synergistic effect of plasmonic metal NPs–CNTs in mesoporous wide bandgap semiconductors sensitized with QDs on the light harvesting efficiency and the carrier transport/collection efficiency is as yet not understood. In addition, the optimization of plasmonic metal NPs–CNTs hybrid network is critical to optimize performance in solar energy conversion devices.

Here we demonstrate for the first time a facile synthetic approach for the fabrication of QDs/TiO_2_–Au:CNTs hybrid network based PEC devices for H_2_ generation. We decorated Au NPs on the surface of CNTs by using a simple hydrothermal approach with different relative ratio of Au and CNTs. Subsequently we prepared TiO_2_–Au:CNTs hybrid anodes with different amount (wt%) of Au:CNTs hybrid network using the simple and cost‐effective doctor blade approach. A specially designed colloidal alloyed CdSe/(CdSe*_x_*S_1−_ *_x_*)_5_/(CdS)_1_ QDs “giant” core/shell QDs (denoted as “g‐QD”) with broad absorption as well as efficient exciton separation were applied as light harvester. The PEC device based on “g‐QD” sensitized TiO_2_–Au:CNTs hybrid photoanode with an optimized amount of Au:CNTs (0.10:0.014 wt%) yielded a ≈26% higher saturated photocurrent density than that of the control PEC device. This improvement is attributed to enhanced light harvesting efficiency, hot‐electron injection and near‐field enhancement induced by the Au NPs and the improved electron transport provided by CNTs directional path through the mesoporous film. In addition, the long‐term stability of the PEC device based on a QDs/TiO_2_–Au:CNTs photoanode is better than the PEC devices based on QDs/TiO_2_ photoanode.

## 2 Results and Discussions

### 2.1 Structural Characterization of Photoanode

**Figure 1:**
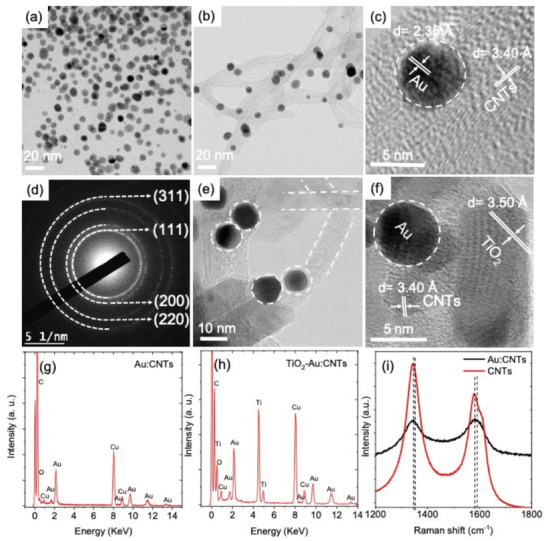
Structural characterization of Au, Au:CNTs, and TiO_2_–Au:CNTs hybrid network: a) as‐synthesized Au NPs; b) Au:CNTs; c) HR‐TEM of Au:CNTs; d) SAED of Au NPs; e) TiO_2_–Au:CNTs; f) HR‐TEM of TiO_2_–Au:CNTs. Elemental analysis of Au:CNTs and TiO_2_–Au:CNTs by EDS: g) Au:CNTs; h) TiO_2_–Au:CNTs. i) Raman spectra of CNTs (red color line) and Au:CNTs (black color line).

**Figure** **1**a displays a transmission electron microscopy (TEM) image of the as‐synthesized polyethyleneimine (PEI) coated Au NPs with spherical shape and an average size of 7.56 ± 0.05 nm. The size distribution of as‐synthesized PEI coated Au NPs is shown in Figure S1a in the Supporting Information. Figure S1b in the Supporting Information displays the absorption spectrum of PEI‐coated Au NPs with a surface plasmon absorption band located at 535 nm. The hybrid network of Au:CNTs was prepared by mixing a precise amount of aqueous Au NPs solution into ethanolic suspension of CNTs via a simple hydrothermal approach. PEI coated Au NPs were attached to the sidewall of the entire CNTs via noncovalent electrostatic interaction (Figure 1b). A detailed description of the nature of bonding between Au and CNTs is discussed below by using Raman measurements. High‐resolution‐TEM (HR‐TEM) image of the Au:CNTs hybrid network is shown in Figure 1c. The interplanner spacing of Au NPs is around 2.35 Å, which corresponds to (111) planes of face centered cubic phase of Au (JPCD file No: 00‐004‐0784), whereas the distance between the adjacent graphene layers within CNTs is around 3.4 Å, consistent with previous reports.^[^ ^22^, ^27^ ^]^ Selected area electron diffraction (SAED) pattern of Au NPs is displayed in Figure 1d, composed of several concentric rings corresponding to (111), (200), (220) and (311) planes of the face‐centered cubic phase structure of Au (JPCD file No: 00‐004‐0784).

Furthermore, energy‐dispersive X‐ray spectroscopy (EDS) measurements of the Au:CNTs hybrid network confirms the presence of Au and C (from CNTs) (Figure 1g).

The Au:CNTs hybrid network solution was then mixed with an appropriate amount of TiO_2_ paste and deposited on the fluorine doped tin oxide (FTO) by using doctor blade approach followed by annealing at 500 °C for 30 min. Figure 1e shows a TEM image of TiO_2_–Au:CNTs hybrid anode with Au NPs highlighted by white dotted circles and CNTs by white dotted lines. The HR‐TEM of the TiO_2_–Au:CNTs hybrid anode shown in Figure 1f with interplanar spacing of 3.50 Å for TiO_2_ confirms the anatase phase of TiO_2_ and 3.40 Å for CNTs and 2.35 Å for Au. There is no change in the size, morphology, and crystal structure of both Au NPs and CNTs during the preparation of TiO_2_–Au:CNTs hybrid anode, confirming the thermal and structural stability of CNTs and Au NPs. Figure 1h displays the EDS spectra of TiO_2_–Au:CNTs hybrid anode, which confirms the presence of Au, C (from Au:CNTs) and Ti, O (from TiO_2_). Raman spectroscopy was used to further investigate the nature of interaction of cationic PEI coated Au NPs with CNTs. Figure 1i depicts the Raman spectra of CNTs and Au:CNTs hybrid network with relative ratio of 2:1. The D and G bands of the pristine CNTs are located at 1346 and 1578 cm^−1^, respectively. The later bands are most commonly observed peaks for the carbonaceous materials.^[^ ^28^ ^]^ The Raman spectrum of the Au:CNTs hybrid network shows a slightly shift in G bands from 1578 to 1583 cm^−1^, and D band from 1345 to 1347 cm^−1^, which confirms binding of PEI coated Au NPs to CNTs (Figure 1i). The relative ratio of the D and G bands (*I*_D_/*I*_G_) is accepted as an indication of binding at the sidewalls or changes in the defects of CNTs. The *I*_D_/*I*_G_ ratio was reduced from 1.23 to 0.98 for pristine CNTs to Au:CNTs, respectively (see Table S1 in the Supporting Information). This suggests that PEI coated Au NPs are attached to the sidewall of the CNTs via physisorption and electrostatic interaction as expected.^[^ ^29^, ^30^, ^31^, ^32^ ^]^

The morphology of the mesoporous anode is a crucial component for PEC device performance. Scanning electron microscopy (SEM) images of TiO_2_, TiO_2_–CNTs, TiO_2_–Au, and TiO_2_–Au:CNTs hybrid mesoporous film deposited on the FTO glass substrate are shown in Figure S3 in the Supporting Information. The inset of each figure displays a high‐resolution SEM image of the corresponding mesoporous films. The CNTs and Au NPs are hard to distinguish within the hybrid mesoporous films due to the relatively low concentration of CNTs (0.014 wt%) and Au (0.010 wt%) and their conformal coverage by the TiO_2_ NPs. However the TiO_2_–Au:CNTs hybrid mesoporous film exhibits cracks at higher concentration of the Au:CNTs (0.015:0.014) wt% (Figure S3e,f, Supporting Information) thereby hindering electron transport within the mesoporous film and acting as recombination sites for the photogenerated carrier at the electrolyte/FTO interface. The absorption spectra of bare TiO_2_ and TiO_2_–Au, and TiO_2_–Au:CNTs hybrid mesoporous films with different content of Au:CNTs (wt%) are shown in Figure S4 in the Supporting Information. Although the content of Au NPs (0.010 wt%) is quite low and conformal coverage of Au NPs by the TiO_2_ NPs, there is still a small photoabsorption in 500–600 nm range in TiO_2_–Au, and TiO_2_–Au:CNTs hybrid mesoporous films due to the surface plasmonic effect of Au NPs, which is absent in bare TiO_2_.

**Figure 2:**
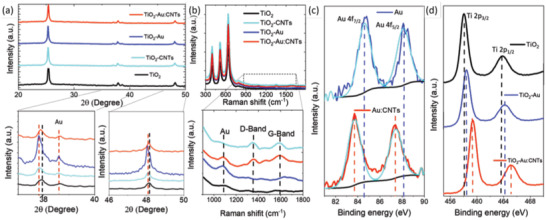
a) XRD patterns of theTiO_2_, TiO_2_–CNTs, TiO_2_–Au, and TiO_2_–Au:CNTs (0.10:0.014 wt%) hybrid mesoporous anodes; b) Raman spectra of mesoporous TiO_2_, TiO_2_–Au, TiO_2_–CNTs, and TiO_2_–Au:CNTs (0.10:0.014 wt%) hybrid anodes and highlights of the D and G bands of CNTs and Au peak (lower image). XPS of: c) Au and Au:CNTs hybrid network; d) TiO_2_, TiO_2_–Au, and TiO_2_–Au:CNTs (0.10:0.014 wt%) hybrid mesoporous anodes.

X‐ray diffraction (XRD) patterns of bare TiO_2_ and TiO_2_–Au, TiO_2_–CNTs and TiO_2_–Au:CNTs hybrid mesoporous films are shown in **Figure** **2**a. There are three peaks located at 2*θ* = 25.3°, 37.9°, and 48.1° of bare TiO_2_ and TiO_2_–Au, TiO_2_–CNTs, and TiO_2_–Au:CNTs hybrid mesoporous films, which correspond to the (101), (004) and (200) crystal planes of TiO_2_ (JCPDS No. 21‐1272). This confirms the anatase phase of TiO_2_ and TiO_2_–Au:CNTs hybrid mesoporous anodes and consistent with previous work. However, it is difficult to distinguish the characteristic peak of CNTs (2*θ* = 26.0°) from the TiO_2_–CNTs and TiO_2_–Au:CNTs hybrid mesoporous anodes due to the overlap with the characteristic peak of anatase TiO_2_ (2*θ* = 25.3°). There is a characteristic peak of Au NPs observed at 2*θ* = 38.6° both for TiO_2_–Au and TiO_2_–Au:CNTs hybrid anodes corresponding to (111) plane, which is consistent with SAED measurements of Au NPs and confirms the presence of Au NPs.

Figure 2b displays the comparison of Raman spectra acquired from TiO_2_, TiO_2_–Au, TiO_2_–CNTs, and TiO_2_–Au:CNTs hybrid photoanodes. There is a peak at 1120 cm^−1^, which corresponds to the presence of Au NPs in TiO_2_–Au hybrid photoanode (lower image). Similarly, the presence of CNTs in TiO_2_–CNTs hybrid photoanode is confirmed by the D and G bands at 1350 and 1580 cm^−1^, respectively, which are the specific bands position for the carbonaceous materials.^[^ ^28b^ ^]^ The Raman spectra of TiO_2_–Au:CNTs hybrid anodes show the Au peak at 1120 cm^−1^ and the D and G bands at 1350 and 1580 cm^−1^, respectively, which indicate the presence of Au and CNTs. These results demonstrate that the Au NPs and CNTs did not undergo any structural changes while annealing the TiO_2_–Au, TiO_2_–CNTs, and TiO_2_–Au:CNTs hybrid photoanodes at 500 °C for 30 min under ambient conditions.^[^ ^24^, ^26^ ^]^ The relative peak intensities of the D and G bands are lower compared to the same peaks before annealing. This lower intensity of D and G bands is due to a small amount of Au:CNTs hybrid network in TiO_2_ NPs mesoporous film and conformal coverage of CNTs by TiO_2_ NPs.

X‐ray photoelectron spectroscopy (XPS) spectra of the Au NPs and Au:CNTs hybrid network are shown in Figure 2c. The XPS spectra of Au 4f of Au NPs includes two peaks, Au 4f_7/2_ and f_5/2_ at 84.6 and 88.2 eV, respectively. A slight shift was found in the Au 4f characteristic peaks toward lower binding energy (BE) in the Au:CNTs hybrid network such as Au 4f_7/2_ and f_5/2_ at 83.8 and 87.4 eV, respectively. This shift confirms the interaction between the Au NPs and CNTs and the metallic nature of the Au NPs.^[^ ^22^, ^33^ ^]^ At the same time, N 1s BE peaks of Au–PEI, which located at 400.06 and 401.74 eV with a ratio of 13.28 are slightly shifted to 400.43 and 401.97 eV with a ratio of 4.93 in the case of the Au:CNTs hybrid network (Figure S2, Supporting Information). A higher BE peak is usually associated with protonation and/or binding, so decreasing ratio suggests a significant electron donation from N to CNTs (Table S2, Supporting Information). This observation is consistent with the Raman measurement of Au:CNTs hybrid network (Figure 1i). The XPS spectra of Ti 2p can be deconvoluted into two main peaks, Ti 2p_3/2_ and Ti 2p_1/2_. Figure 2d displays the comparison of XPS spectrum of Ti 2p for bare TiO_2_, TiO_2_–Au and TiO_2_–Au:CNTs hybrid mesoporous films. The Ti 2p_3/2_ and Ti 2p_1/2_ peaks of bare TiO_2_ are found at 458.1 and 463.9 eV, respectively, consistently with the previous work.^[^ ^34^ ^]^ With the incorporation of Au NPs in TiO_2_, the Ti 2p peaks slightly shifted toward higher BE; Ti 2p_3/2_ and Ti 2p_1/2_ at 458.3 and 464.2 eV, respectively, compared to bare TiO_2_. Similarly, the spectra from the TiO_2_–Au:CNTs hybrid mesoporous film shows a shift in Ti 2p toward high BE; Ti 2p_3/2_ and Ti 2p_1/2_ at 459.1 and 465.1 eV, respectively. This is attributed to the transfer of electron between Au and TiO_2_^[^ ^35^ ^]^ and the interaction between CNTs and TiO_2_.^[^ ^34^ ^]^

### 2.2 Structural and Optical Characterizations of “g‐QD” and QD Sensitized Anode

**Figure 3:**
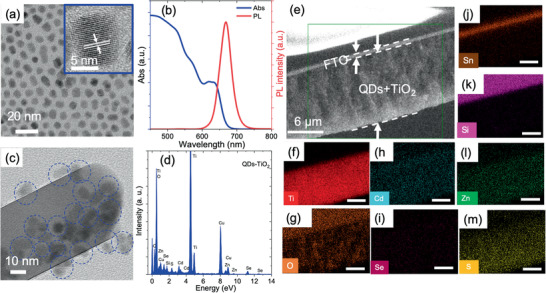
Structural and optical characterizations of colloidal “g‐QDs:” a) TEM image and HR‐TEM (inset); b) absorption and PL spectrum of as‐synthesized QDs in toluene; c) HR‐TEM image of QD sensitized TiO_2_, dotted blue circles highlight the presence of QDs. d) EDS spectrum of “g‐QD” sensitized TiO_2_ coated with ZnS and SiO_2_. e) Cross‐sectional SEM image of QD sensitized TiO_2_ photoanode. EDS mapping of all the elements in ZnS/QDs/TiO_2_ photoanode including: f) Ti; g) O; h) Cd; i) Se; j) Sn; k) Si; l) Zn; m) S (scale bar in (f)‐(m) is 5 µm).

Colloidal “g‐QDs” were synthesized according to a previous report.^[^ ^26^ ^]^ In brief, CdSe core QDs with average diameter of 3.33 ± 0.02 nm were synthesized via a hot injection approach. A typical TEM image and size distribution of as‐synthesized CdSe core QDs are shown in Figure S5a,b in the Supporting Information. CdSe*_x_*S_1−_ *_x_* graded alloyed interfacial layers were further grown over the CdSe core QDs by tailoring the Se:S ratio during in situ growth of each CdSe*_x_*S_1−_ *_x_* (*x* = 0.9–0.1) via a successive ionic layer adsorption and reaction (SILAR) at 240 °C under N_2_ flow. The final diameter of the “g‐QDs” after the growth of five graded alloyed interfacial layers of CdSe*_x_*S_1−_ *_x_* (*x* = 0.9–0.1) with one monolayer of CdS reaches 7.75 ± 0.10 nm (estimated from the TEM image) (**Figure** **3**a).

Figure S5c in the Supporting Information shows the size distribution of the “g‐QDs.” The inset of Figure 3a displays an HR‐TEM image of “g‐QDs” with clear lattice fringes confirm the high crystallinity of QDs. The calculated interplanar spacing of QDs is 3.60 Å, corresponding to (101) plans of the Wurtzite (WZ) crystal structure. Figure S5d in the Supporting Information displays the EDS spectra of “g‐QDs,” confirming the presence of Cd, Se and S elements. The SAED pattern of QDs is shown in Figure S5e in the Supporting Information, which corresponds to the (111), (211), (220), (310), and (311) planes of the WZ phase, respectively, and further supported by XRD pattern of “g‐QDs.” The peak positions of the XRD pattern show the combined reflection of the WZ crystal structure of CdS and the WZ crystal structure of CdSe (Figure S5f, Supporting Information), which confirms the formation of graded alloyed QDs.^[^ ^36^ ^]^ Figure 3b displays the absorption and photoluminescence (PL) spectrum of as‐synthesized “g‐QDs” in toluene. The first‐excitonic absorption peak was observed around 633 nm with broad absorption spectral range from 400 to 680 nm, which is required for the realization of efficient PEC devices. The PL peak of “g‐QDs” was observed at 668 nm (Figure 3b).

There are several approaches to anchor the colloidal QDs onto the TiO_2_ mesoporous film, such as bilinker and electrophoretic deposition (EPD).^[^ ^37^ ^]^ In the bilinker approach, a bifunctional molecule is linked between the QDs to surface TiO_2_ NPs mesoporous film and the electron transport rate at the QD/TiO_2_ interface is directly related to the length of bifunctional molecule.^[^ ^38^ ^]^ In the EPD approach, colloidal QDs are directly anchored to surface of the TiO_2_ NPs mesoporous film, yielding enhanced electron transfer rate compared to bilinker approach as confirmed in our previous reports.^[^ ^12^, ^38^ ^]^ Therefore, we used the EPD approach to deposit the as‐synthesized graded alloyed core/shell QDs into the TiO_2_ NPs mesoporous film. To verify the homogenous distribution of colloidal QDs into the TiO_2_ mesoporous film, we carried out EDS measurements. Figure 3e displays the cross‐sectional SEM of the QD sensitized TiO_2_ NPs mesoporous film and EDS mapping of the selected green rectangular area are reported in Figure 3f–m. The EDS mapping and spectra of the QD sensitized TiO_2_ NPs mesoporous film coated with ZnS and SiO_2_ confirm the homogeneous distribution of “g‐QDs” within the TiO_2_ NPs mesoporous film and confirm the presence of Ti, O, Cd, Se, S, Zn, and Si. In addition, homogenous dispersion of the colloidal “g‐QDs” on the surface of the TiO_2_ NPs is also confirmed from the HR‐TEM images of QD sensitized TiO_2_ NPs mesoporous film. Figure 3c displays the HR‐TEM image of QD (blue dotted circles) sensitized TiO_2_ NPs mesoporous film, which confirms that there is no aggregation of QDs during their deposition via EPD. The EDS spectra of “g‐QD” sensitized TiO_2_ NPs mesoporous film coated with ZnS and SiO_2_ also confirm the presence of Ti, O, Cd, Se, S, Zn and Si (Figure 3d).

### 2.3 PEC Performance

**Figure 4:**
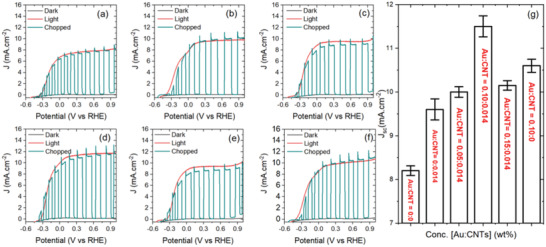
Photocurrent density versus potential (vs RHE) of PEC device based colloidal “g‐QD” sensitized TiO_2_–Au:CNTs hybrid photoanodes with different relative ratio concentrations (wt%) of Au and CNTs under dark, chopped, and continuous 1 sun light illumination (AM 1.5G, 100 mW cm^−2^); a) 0:0; b) 0:0.014; c) 0.05:0.014; d) 0.10:0.014; e) 0.15:0.014; f) 0.10:0.0. g) Variation of the saturated photocurrent density with the relative concentration ratio of Au and CNTs at 1.0 V versus RHE.

Solar‐driven PEC devices for hydrogen generation were fabricated by using colloidal “g‐QD” sensitized TiO_2_ and TiO_2_–Au:CNTs hybrid mesoporous film with different amounts (wt%) of Au:CNTs hybrid network as working electrode to highlight the effect of Au:CNTs. The current density measurements were carried out under dark, chopped and continuous 1 sun light illumination (AM 1.5G, 100 mW cm^−2^) by using a typical three electrodes configuration system with Platinum as the counter electrode and Ag/AgCl as the reference electrode. **Figure** **4**a–h displays the photocurrent density versus potential [vs reversible hydrogen electrode (RHE)] curves of PEC device based QDs/TiO_2_–Au:CNTs hybrid photoanodes with different relative concentration of Au and CNTs. The calculated saturated photocurrent densities of all PEC devices at 1.0 V versus RHE are reported in **Table** **1**. The photocurrent densities of all devices increase with the addition of Au:CNTs hybrid network in TiO_2_ mesoporous film with varying weight ratios of Au:CNTs (0.0:0.0 to 0.15:0.014 wt%). In brief, the highest saturated photocurrent density of the PEC device based on QDs/TiO_2_ photoanode under 1 sun illumination (100 mW cm^−2^) is 8.20 ± 0.11 mA cm^−2^ (Figure 4a), which increases to 9.60 ± 0.24 mA cm^−2^ upon addition of Au:CNTs (0.0:0.014) wt% due to enhanced electron transport and collection efficiency of the photoinjected electrons by the directional path of CNTs as confirmed in our previous report (Figure 4b).^[^ ^26^ ^]^ Further the incorporation of Au NPs decorated CNTs with concentration ratio of 0.05:0.014 wt%, enhances the saturated photocurrent density to 10.00 ± 0.12 mA cm^−2^ (Figure 4c), then reaches to a maximum value of 11.50 ± 0.24 mA cm^−2^ with increase of Au NPs content (0.10 wt%), while maintaining the same content (0.014 wt%) of CNTs, which is ≈40% higher than that of the PEC device based on the bare QDs/TiO_2_ photoanode (Figure 4d). This significant improvement in the photocurrent density of PEC device with the addition of precise amount of Au:CNTs in the QDs/TiO_2_ is mainly attributed to the synergetic effect of the large light absorption cross‐section of the Au NPs, that contribute to the charge collection efficiency by enhancing the absorption of the “g‐QDs” and directly injecting hot electrons to the supporting structures, with improved electron transport and collection provided by CNTs directional path to the photogenerated electrons through the TiO_2_ mesoporous film (see carrier dynamic section for more details). In addition, the presence of this small amount of Au NPs (0.10 wt%) decorated CNTs (0.014 wt%) in TiO_2_ NPs mesoporous film does not alter the structural morphology and optical transparency, which is confirmed by SEM images (Figure S3, Supporting Information) and UV–visible absorption (Figure S3, Supporting Information).

However, if we further increase the concentration of Au:CNTs (0.15:0.014 wt%), the saturated photocurrent density decreases from 11.50 ± 0.24 to 10.15 ± 0.11 mA cm^−2^ (Figure 4e). This is mainly attributed to the crack formation with the addition of high concentration of Au:CNTs (0.15:0.014 wt%) in TiO_2_ (Figure S3, Supporting Information), which act as recombination center during device operation and reduce overall electron transport. In addition, it starts partially absorbing the light that is available for exciton generation, which reduces the overall performance of PEC devices. To highlight the effect of Au NPs, the PEC device was also fabricated using QDs/TiO_2_–Au with 0.10 wt% of Au NPs in TiO_2_, yielding a saturated photocurrent density of 10.60 ± 0.15 mA cm^−2^ under 1 sun illumination, which is ≈29% and ≈10% higher than that of the PEC devices based on QDs/TiO_2_ and QDs/TiO_2_–CNTs (0.014 wt%), respectively. The variation of the saturated photocurrent density of PEC devices based on QDs/TiO_2_–Au:CNTs hybrid photoanodes with varying content (wt%) of Au NPs and CNTs at 1.0 V versus RHE under 1 sun illumination is shown in Figure 4g and values are reported in Table 1.

**Table 1:** Calculated photocurrent density of PEC devices based on QDs/TiO_2_–Au:CNTs with varying weight ratios of Au:CNTs (0.0:0.00 to 0.15:0.014 wt%) in TiO_2_ at 1.0 V versus RHE under 1 sun illumination (AM 1.5G, 100 mW cm^−2^)

Anode structure	Au:CNT [wt%]	*J* [mA cm^−2^]
TiO_2_	0.00:0.00	8.20 ± 0.11
TiO_2_–Au:CNTs	0.00:0.014	9.60 ± 0.24
	0.05:0.014	10.00 ± 0.12
	0.10:0.014	11.50 ± 0.24
	0.15:0.014	10.15 ± 0.11
	0.10:0.00	10.60 ± 0.15

Thus, the PEC device based on the QDs/TiO_2_–Au:CNTs photoanodes with Au:CNTs of 0.10:0.014 wt% yields the highest saturated photocurrent density compared to all other types of QDs/TiO_2_–Au:CNTs photoanodes with different relative content (wt%) of Au and CNTs.

### 2.4 Carrier Dynamics in the Hybrid Anode

**Figure 5:**
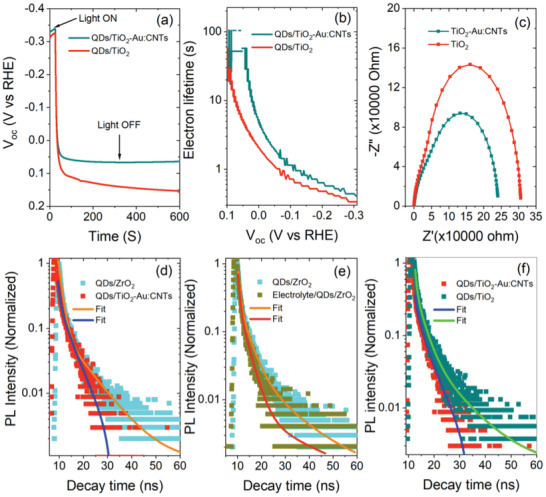
Transient photovoltage decay measurements of PEC devices based on QDs/TiO_2_–Au:CNTs and QDs/TiO_2_ photoanodes: a) *V*_oc_ decay; b) electron lifetime calculated from the *V*_oc_ decay. c) Nyquist plot of PEC devices based on TiO_2_–Au:CNTs and TiO_2_ at 0.6 V applied bias in three electrode configuration under dark conditions. Comparison of the transient PL curves: d) QDs/ZrO_2_ and QDs/TiO_2_–Au:CNTs mesoporous film; e) QDs/ZrO_2_ mesoporous film and electrolyte/QDs/ZrO_2_; f) QDs/TiO_2_–Au:CNTs and QDs/TiO_2_ mesoporous film. The excitation wavelength is  *λ* = 444 nm.

To gain further insights into the carrier transport properties of QDs/TiO_2_–Au:CNTs hybrid and QDs/TiO_2_ photoanodes, we used transient photovoltage decay spectroscopy. To measure the transient photovoltage decay, both PEC devices based on QDs/TiO_2_–Au:CNTs hybrid and QDs/TiO_2_ photoanodes were illuminated under 1 sun simulated sunlight (AM 1.5G, 100 mW cm^−2^) until reaching a steady voltage and then shut down and recoded the voltage decay with time under dark conditions. **Figure** **5**a displays the open‐circuit voltage (*V*_oc_) response of PEC devices based on QDs/TiO_2_ and QDs/TiO_2_–Au:CNTs hybrid photoanodes under illumination followed by measurements without illumination. Under illumination, the photogenerated electron injected to the conduction band (CB) of TiO_2_ from the photoexcited QDs leads to the shift of the Fermi level to more negative potential and enhances the *V*_oc_. When the electron accumulation equal to charge recombination and then the *V*_oc_ value reaches to the maximum value and attains a steady state. Under dark conditions, the *V*_oc_ decay rate corresponds to the recombination of the accumulated electrons with electron acceptor in the electrolyte and within the TiO_2_ network.^[^ ^39^ ^]^

The *V*_oc_ rate is faster for the PEC device based on QDs/TiO_2_ photoanodes than the PEC device based on QDs/TiO_2_–Au:CNTs hybrid photoanodes (Figure 5a). The electron lifetime (*τ*_e_) of the corresponding PEC devices was calculated from *V*_oc_ decay rate by using the following equation^[^ ^40^ ^]^

(1) $$\begin{array}{c} \tau_{e}=\left(\frac{k_{B}T}{e}\right){\left(\frac{dV_{\mathrm{oc}}}{dt}\right)}^{-1} \end{array}$$

where *k*_B_ is Boltzmann's constant, *T* is the absolute temperature, and *e* is the electron's charge.

The *τ*_e_ depends on the nonradiative carrier recombination at the photoanode/electrolyte interface. The calculated *τ*_e_ value is higher for PEC devices based on QDs/TiO_2_–Au:CNTs hybrid photoanode than that of PEC devices based on the QDs/TiO_2_ photoanode (Figure 5b). The higher value of *τ*_e_ for PEC device with QDs/TiO_2_–Au:CNTs hybrid photoanode confirms the lower carrier recombination compared to PEC with QDs/TiO_2_. Thus, the incorporation of Au nanoparticle decorated CNTs hybrid network in TiO_2_ suppresses the nonradiative carrier recombination due to directional path offered by CNTs and enhances charge extraction by plasmonic effect of Au NPs due to improved exciton generation in QDs. In addition, the surface traps near the lower edge of the CB edge reduces with the incorporation of Au:CNTs hybrid network in TiO_2_, which in turn decreases the interfacial carrier recombination rate.^[^ ^41^ ^]^

In addition, EIS measurements were carried out to understand the effect of Au:CNTs hybrid network on the carrier dynamics within TiO_2_ mesoporous film under dark conditions. In this measurement, the three electrode configuration was used: Pt as counter electrode; Ag/AgCl as reference and TiO_2_–Au:CNTs or TiO_2_ photoanode as a working electrode (Figure S6a, Supporting Information). An equivalent circuit composed of the series resistance (*R*_s_) followed by the series of the charge transfer resistance (*R*_ct_) and Warburg element and then parallel by a constant phase element (CPE) was used to fit the mid‐frequency range data (Figure S6b, Supporting Information). Figure 5c displays the Nyquist plots of the PEC devices based on TiO_2_–Au:CNTs and TiO_2_ photoanodes. The semicircle of the Nyquist plots defines the carrier dynamics at the photoanode/electrolyte interface. The diameter of the semicircle for a PEC device with TiO_2_–Au:CNTs is smaller than that of PEC device with TiO_2_, which confirms reduced *R*_ct_ for the photogenerated carrier in TiO_2_–Au:CNTs. Thus the incorporation of a precise amount of Au:CNTs hybrid network (0.10:0.014 wt%) in TiO_2_ mesoporous film reduces the *R*_ct_ compared to TiO_2_ as well. The *R*_ct_ values of PEC devices based on TiO_2_–Au:CNTs and TiO_2_ photoanodes were calculated by fitting the Nyquist plots at different bias potential versus RHE. At 0.6 V versus RHE, *R*_ct_ of the PEC device based on the TiO_2_–Au:CNTs (*R*_ct_ = 6.3 kΩ) is lower than that of PEC device based on TiO_2_ (*R*_ct_ = 9.9 kΩ), which confirm the better electron transport and injection rate in TiO_2_–Au:CNTs compared to the TiO_2_ photoanode.

In addition, we used transient PL spectroscopy to investigate the carrier dynamics of QDs coupled with TiO_2_–Au:CNTs hybrid and TiO_2_ anodes as an electron and electrolyte containing 0.25 m sodium sulfide (Na_2_S) and 0.35 m sodium sulfite (Na_2_SO_3_) (pH = 13) as hole scavengers. QD/ZrO_2_ photoanodes were used as a reference, in which there is no electron transfer from QDs to ZrO_2_ due to the unfavorable electronic band alignment of QDs and ZrO_2_. For efficient carrier transfer of the photogenerated carriers in the QDs to their scavengers depends on the optimization of the band alignment of the conduction band edge of the QDs with the conduction band edge of the metal oxide (TiO_2_–Au:CNTs hybrid and TiO_2_ anodes) and the redox potential of the sulfur‐based electrolyte with the valence band of the QDs. In our previous reports, we confirmed that there is no possibility of the energy transfer from the QDs to the carrier scavengers as there is no spectral overlap between the PL of the QDs and absorption of the carrier scavengers.^[^ ^42^ ^]^ The PL decay of the QDs was measured under pulse laser of 444 nm and all curves were fitted using an exponential decay. The average lifetime (〈*τ* 〉) was extracted by using the following equation

(2) $$\begin{array}{c} 〈\tau 〉=\frac{a_{1}\tau_{1}^{2}+a_{2}\tau_{2}^{2}+a_{3}\tau_{3}^{2}}{a_{1}\tau_{1}+a_{2}\tau_{2}+a_{3}\tau_{3}} \end{array}$$

where *a_i_* (*i* = 1, 2, 3) are the fitting coefficients of the PL decay and *τ*_*i*_ (*i* = 1, 2, 3) are the characteristic lifetimes, respectively.

Figure 5d–f displays the comparison of PL decay rate of QDs/ZrO_2_, electrolyte/QDs/ZrO_2_, QDs/TiO_2_–Au:CNTs and QDs/TiO_2_. These results demonstrate that the PL decay is faster for QDs/TiO_2_–Au:CNTs than the QDs/ZrO_2_ due to different band offset potential between the QDs and ZrO_2_ or TiO_2_–Au:CNTs. This confirms the efficient transfer of the electrons from the QDs to the TiO_2_–Au:CNTs with suitable band alignment. Similarly, the PL decay of QDs with ZrO_2_/electrolyte is faster than only ZrO_2_ due to efficient transfer of the holes from QDs to the electrolyte in suitable band alignment of the QDs with the redox potential of the electrolyte (Figure 5e). To highlight the effect of the Au:CNTs hybrid network, we compared the PL decay rate of the QDs/TiO_2_–Au:CNTs and QDs/TiO_2_ (Figure 5f). The PL decay rate is slightly faster for the QDs/TiO_2_–Au:CNTs than that of the QDs/TiO_2_. The calculated average electron lifetime is 8.8 ± 0.02 and 12.0 ± 0.08 ns for QDs/TiO_2_–Au:CNTs and QDs/TiO_2_, respectively. This reduced lifetime value (38%) is mainly due to enhanced carrier extraction and injection kinetics by plasmonic‐electrical effect of Au NPs. In QDs/TiO_2_–Au:CNTs hybrid system, Au NPs induce the local field enhancement that improves not only light absorption, but also leads to better carrier separation and transport. In addition, the improved injection kinetics is also attributed to slight negative shift in the CB minimum (CBM) with the incorporation of Au:CNTs hybrid network in TiO_2_ as confirmed by the ultraviolet photoelectron spectroscopy (UPS) measurements.

The carrier transfer rates (*K*_et_ or *K*_ht_) of QDs coupled with the different carrier scavengers were calculated from the difference in the lifetime values of the corresponding carriers, by using the following equation

(3) $$\begin{array}{ccc} K_{\mathrm{et}\;\mathrm{or}\;\mathrm{ht}} & = & \frac{1}{\left(〈\tau 〉\mathrm{QDs}/e\left(\mathrm{Ti}O_{2}-\mathrm{Au}:\mathrm{CNTs}\right)\mathrm{or}\;h\left(\mathrm{electrolyte}\right)\right)}\\ & & -\frac{1}{〈\tau 〉\mathrm{QDs}/\left(\mathrm{Zr}O_{2}\right)} \end{array}$$

where 〈*τ*〉QDs/*e*(TiO_2_ − Au: CNTs) or *h*(electrolyte) and 〈*τ*〉QDs/ZrO_2_ are the average lifetimes of the QDs with carrier scavengers and QDs with ZrO_2_, respectively. The calculated electron transfer rate for QDs/TiO_2_–Au:CNTs (*K*_et_ = 4.4 ± 0.8 × 10^7^ s^−1^) is faster than that of QDs/TiO_2_ (1.4 ± 0.08 × 10^7^ s^−1^) and hole transfer rate (*K*_ht_ = 2.1 ± 0.05 × 10^7^ s^−1^) values, confirming efficient transfer from the QDs to the carrier scavengers (TiO_2_–Au:CNTs and electrolyte).

Ultravoilet photoelectron spectroscopy (UPS) spectra were acquired to further investigate the effect of the incorporation of Au:CNTs hybrid network on the electronic bandgap alignment of TiO_2_. The energy bandgap values of TiO_2_ and TiO_2_–Au:CNTs were calculated from their absorption spectra by using the classical Tauc plots. The bandgap of TiO_2_ is found to be 3.25 eV, which is consistent with its known value,^[^ ^43^ ^]^ whereas the bandgap of TiO_2_–Au:CNTs is 3.02 eV (Figure S7a, Supporting Information). UPS with He I radiation (21.22 eV) was used to estimate the Fermi level and valance band maximum (VBM) level energy of TiO_2_ and TiO_2_–Au:CNTs. Figure S7b in the Supporting Information displays full UPS spectrum of both TiO_2_ and TiO_2_–Au:CNTs with high and low binding energy cut‐off, which determines the Fermi level and VBM level energy, respectively. The CBM level value for TiO_2_ and TiO_2_–Au:CNTs can be calculated from the VBM and bandgap values (Figure S7c,d, Supporting Information). These results show that the bandgap of TiO_2_ is slightly reduced with the incorporation of the Au–CNTs hybrid network consistent with the combined effect of Au^[^ ^44^ ^]^ and CNTs,^[^ ^45^ ^]^ and having negative shift (eV) in the CBM level. This shift has positive effect on electron injection rate from the CB of QDs to TiO_2_–Au:CNTs and suppresses the charge recombination during the device performance, which is consistent with transient PL spectroscopy and photovoltage decay measurements (Figure 5). The schematic diagram with summarized electronic band alignment of TiO_2_ and TiO_2_–Au:CNTs sensitized with alloyed core/shell QDs for PEC cells is shown in Figure 7a).

In summary, the results of the carrier dynamics measurements are consistent with the obtained photocurrent density of PEC devices based on the respective photoanodes.

### 2.5 Theoretical Investigation of Interaction of QDs with TiO_2_–Au:CNTs

Figure 4 shows that the inclusion of Au NPs enhances the overall photocurrent generation at the photoanode. However, this occurs through the joint operation of several distinct physical processes, arising from the interaction of the different material systems present in our device. Here we briefly address some of the relevant mechanisms at play and comment their respective roles.

**Figure 6:**
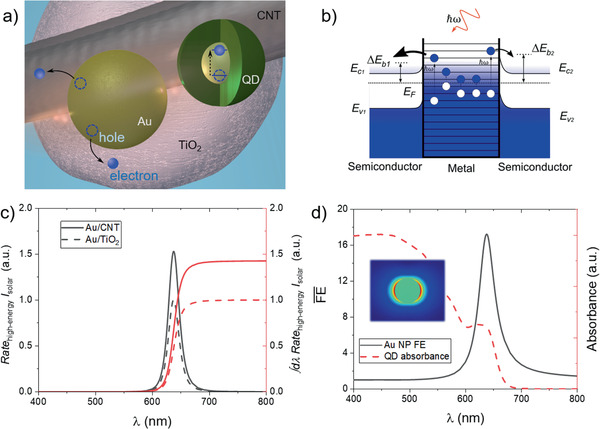
a) Schematic diagram of processes through which the Au NPs contribute to the photocurrent in the device, i.e., hot‐electron injection both to TiO_2_ and CNT and the promotion of electron–hole pair excitation in the QDs through interaction with the greatly enhanced near‐field of the Au NCs. b) Schematic energy diagram of the surface‐driven generation of hot electrons and their injection to two different semiconductors forming interfaces with the metal with different Schottky energy barriers. The lower energy barrier on the left interface leads to a higher injection rate of hot electrons to semiconductor #1 (CNT), represented by the different thickness of the injection arrows in the diagram. c) Rate of injection of hot electrons through two metal/semiconductor interfaces with different Schottky barrier heights. The theoretical data suggest that we should expect a larger injection rate from Au when including CNTs in the TiO_2_ mesoporous system. d) Comparison of the spectral field enhancement of the Au NPs, averaged over a volume surrounding the NP, with the absorbance of the core–shell QDs. The plasmon band strongly overlaps with the energy of the first excited state of the QD, which supports the direct plasmonic enhancement of light absorption at the g‐QDs.

First, let us consider the mechanisms through which the Au NPs contribute to the photocurrent in the absence of QDs (Figure S8, Supporting Information). Given that the plasmon resonance of the small Au NPs surrounded by TiO_2_ occurs at longer wavelengths than those covered by the absorption band of TiO_2_, their main contribution to the photocurrent will be the injection of plasmonic hot electrons (**Figure** **6**a).^[^ ^46^ ^]^ Importantly, we should also contemplate the possibility of the plasmonic hot electrons being injected into the CNTs, which can account for part of the synergistic improvement coming from the addition of both Au NPs and CNTs, even in the absence of g‐QDs (Figure S8, Supporting Information). Briefly, the mechanism responsible for introducing these additional carriers into the generated photocurrent proceeds in two steps, carrier excitation in the metal and injection through the metal–semiconductor interface (Figure 6b). However, most of the plasmon‐excited carriers will have a small amount of additional energy and will therefore populate states close to the Fermi energy of the metal. Only electrons with energies larger than the Schottky energy barrier at the metal–semiconductor interface will traverse it and contribute to the total photocurrent. These high‐energy, hot electrons represent a relatively low proportion of the total charge carriers excited in the metal, and their generation requires a substantial change in the linear momentum of the electron.^[^ ^46^, ^47^ ^]^ This is favored at the surfaces of the NPs, both through the interaction of the electrons with the boundaries of the metal and the large field gradient that can appear in certain NP geometries.^[^ ^48^ ^]^

We can calculate the number of high‐energy hot electrons generated in a given plasmonic nanostructure by using the formalism described in refs. [48, 49], which quantifies the surface‐driven generation of high‐energy carriers. The relevant expression is the following

(4) $$\begin{array}{c} \mathrm{Rat}e_{\mathrm{high}-\mathrm{energy}}=\frac{1}{4}\frac{2}{\pi^{2}}\frac{e^{2}E_{F}^{2}}{\hbar }\frac{\left(\hbar \omega -\Delta E_{b}\right)}{{\left(\hbar \omega \right)}^{4}}\int_{S_{\mathrm{NC}}}{\left|E_{\omega ,\mathrm{normal}}\left(\theta ,\varphi \right)\right|}^{2}ds \end{array}$$

and it permits the calculation of the rate of generation of electrons with energies higher than the energy barrier Δ*E*_b_. This magnitude, with units of 1/*s*, also depends on the Fermi energy of the metal, *E*_F_, the photon's energy, ℏ*ω*, and the integral over the NPs surface, *S*_NC_, of the squared absolute value of the complex amplitude of the field normal to the inner surface of the metal particle.

In our case, the injection of plasmonic hot electrons occurs at the interfaces with two different semiconducting systems, TiO_2_ and CNTs. In the context of this model, these two semiconductors will induce different injection rates by virtue of their different Schottky barrier heights when interfacing with the Au NPs. Thus, and as schematically depicted in Figure 6b, the interface with the lower barrier will experience a larger flux of charge carriers. The data in Figure 6c presents results obtained with Equation (4), for the two different cases. The Au/TiO_2_ interface is modelled with Δ*E*_b_ = 1 eV,^[^ ^50^ ^]^ while the Au/CNT is estimated as Δ*E*_b_ = 0.5 eV.^[^ ^51^ ^]^ A lower energy barrier at the Au/CNT interface would thus lead us to expect an increase in the plasmon‐induced photocurrent if we compare a system including CNTs with one in which the Au NPs only interacts with TiO_2_. The quantitative expression of this concept is displayed in Figure 6c.

In the case of the system sensitized with QDs, the previous perspective still holds, but the injection of plasmonic hot electrons into TiO_2_ and CNTs is greatly surpassed in magnitude by the sensitization of the g‐QDs. Nonetheless, the data in Figure 4 clearly shows that the presence of the Au NPs enhances the overall efficiency of the device. To understand this observation, we now discuss the interaction between the Au NPs with the g‐QDs. One of the central properties of plasmonic resonances is their enhancement of the electric field near the NPs supporting them. In turn, the intense near‐field drives secondary optical processes in adjacent structures, with the relevant phenomenon in this case being the promotion of electron–hole pairs in the g‐QDs.^[^ ^21^, ^52^ ^]^ This contribution to the QD photoabsorption can be interpreted as the Au NPs increasing the photonic density of states at the neighboring QDs at the Au NPs’ resonant frequency, thus increasing the probability of light absorption by the QDs. Supporting the interpretation that this is what occurs in our QD‐sensitized photoanode, we show that the plasmon band of 10 nm Au NPs immersed in mesoporous TiO_2_ overlaps with the absorption spectrum of the QDs. This is depicted in Figure 6d, where we compare the spectrum of the plasmonic field enhancement around the Au NPs with the experimental absorbance of the core–shell QDs used in this study. Particularly, we calculate the average field enhancement as

(5) $$\begin{array}{c} {\bar{\mathrm{FE}}}_{\mathrm{ext}}=\frac{1}{V}\int_{V}dr^{3}\frac{{\left|E\left(r\right)\right|}^{2}}{E_{0}^{2}} \end{array}$$

where *V* is the volume of the space surrounding the Au NP (a virtual “shell” with a thickness of 10 nm surrounding the NP), $E_{0}$ is the electric field amplitude of the incoming radiation, and **E**(**r**) is the electric field at a given point in space **r**. Finally, the Au NPs in this system do not contribute to increasing the photon travel path in the photoanode via scattering events, as it is the case for other designs aiming to optimize solar capture.^[^ ^21c^ ^]^ This is so because the plasmon excited in small NPs decay almost exclusively nonradiatively, as shown by results on the interaction cross sections of 10 nm Au NPs in TiO_2_ (Figure S9, Supporting Information).

### 2.6 Superior Performance of PEC

**Figure 7:**
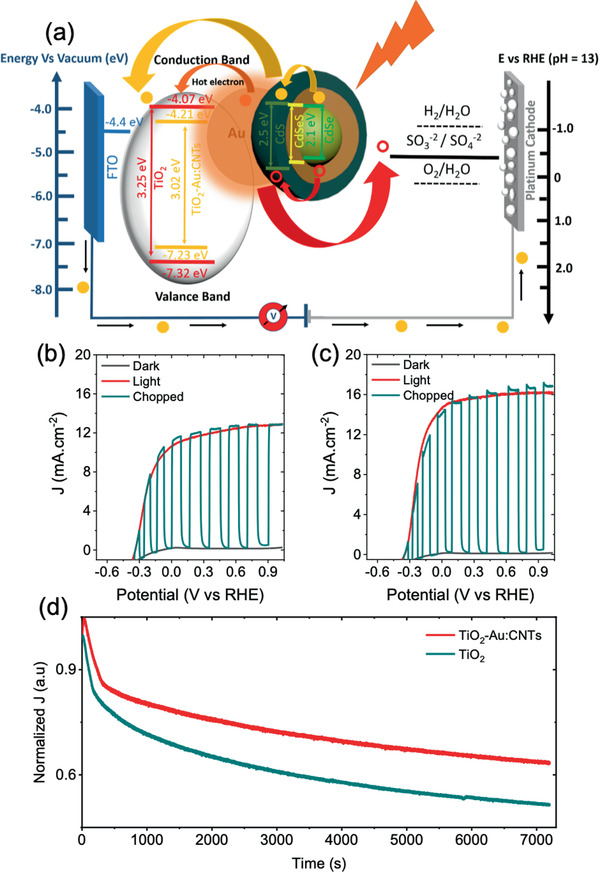
a) Schematic of PEC device with carrier transport and electronic band alignment of TiO_2_ and TiO_2_–Au:CNTs hybrid anode sensitized with alloyed core/shell QDs, calculated from the UPS measurements. Photocurrent density versus potential (vs RHE) of PEC devices under dark, chopped, and continuous 1 sun light illumination (AM 1.5G, 100 mW cm^−2^): b) QDs/TiO_2_; c) QDs/TiO_2_–Au:CNTs. d) Comparison of normalized photocurrent density (a.u.) versus time curves of PEC devices based on QDs/TiO_2_–Au:CNTs and QDs/TiO_2_ at 0.6 V (vs RHE) under 1 sun continuous illumination (AM 1.5G, 100 mW cm^−2^).

To further improve the performance of PEC devices, the photoanode architecture was optimized by introducing two additional layers (details reported in our previous work^[^ ^26^ ^]^): i) 300 nm thin layer of 20 nm sized TiO_2_ NPs spin‐coated between the blocking and active layer; ii) 4–5  µm scattering layer of 150–200 nm sized TiO_2_ NPs deposited over the active layer. The PEC device based on QDs/TiO_2_–Au:CNTs (0.10:0.014 wt%) and QDs/TiO_2_ photoanodes with spin‐coated and scattering TiO_2_ layer exhibits a higher saturated photocurrent density than that of the respective configuration of photoanodes without spin‐coated and scattering TiO_2_ layers. **Figure** **7**b,c displays the photocurrent density versus potential (vs RHE) curves of PEC device based QDs/TiO_2_ and QDs/TiO_2_–Au:CNTs (0.10:0.014 wt%) hybrid photoanodes with spin‐coated and scattering layers under dark, chopped and continuous 1 sun light illumination (AM 1.5G, 100 mW cm^−2^). The calculated saturated photocurrent density of the respective PEC devices at 1.0 V versus RHE under 1 sun illumination is reported in **Table** **2**. The saturated photocurrent density of the PEC device with spin‐coated and scattering layers increases from 11.60 ± 0.15 to 16.10 ± 0.10 mA cm^−2^ for QDs/TiO_2_–Au:CNTs (0.10:0.014 wt%), which is ≈26% higher than the corresponding values obtained from the control device (QDs/TiO_2_). This significant improvement in the saturated photocurrent densities is mainly attributed to: i) improved adhesion between the compact blocking layer and active layer of TiO_2_ NPs with the addition of spin coated TiO_2_ NPs layer; ii) enhanced light scattering with the addition of TiO_2_ scattering layer, which increases the time span by the light within the active layer and the possibility of better light absorption by the QDs. To the best of our knowledge, the highest saturated photocurrent density of 16.10 ± 0.10 mA cm^−2^ (at 1.0 V vs RHE) under 1 sun illumination (AM 1.5G, 100 mW cm^−2^) obtained in this work is higher than the photocurrent density value reported for PEC devices based on hybrid network of plasmonic NPs and wide‐bandgap semiconductor nanostructures sensitized with QDs. For instance, Gong and co‐workers^[^ ^53^ ^]^ fabricated a PEC device based on TiO_2_ branched nanorod arrays decorated with Au NPs and reported a photocurrent density of 2.32 ± 0.1 mA cm^−2^. Wu and co‐workers^[^ ^54^ ^]^ designed a sandwich‐structured CdS QD sensitized Au–TiO_2_ hybrid nanorod array photoanode and reported the highest photocurrent density of 4.07 mA cm^−2^. Later, Wang and co‐workers^[^ ^20a^ ^]^ fabricated a PEC device by exploring novel Au@TiO_2_/Al_2_O_3_/Cu_2_O (p‐n junction) configuration and reported a photocurrent density of −4.34 mA cm^−2^, which is ≈20 times higher compared to that obtained from a TiO_2_‐P25/Cu_2_O photoelectrode. A significant improvement in photocurrent density is obtained by replacing the TiO_2_ mesoporous film with a novel matchlike ZnO nanostructure decorated with plasmonic Au NPs. The resulting PEC device based on ZnO/Au heterostructures yields a photocurrent density of 9.11 mA cm^−2^, which is much higher than that of the pristine ZnO nanorods array (0.33 mA cm^−2^).^[^ ^55^ ^]^ In addition, our methodology adopted in this work to improve the performance of the PEC device is simple, low‐cost, and large‐area scalable compared to the above‐mentioned literature.

**Table 2:** Calculated photocurrent density of PEC devices based on QDs/TiO_2_–Au:CNTs with varying concentration of Au and CNTs in TiO_2_ at 1.0 V versus RHE under 1 sun illumination (AM 1.5G, 100 mW cm^−2^)

Anode structure	Au:CNT [wt%]	*J* [mA cm^−2^]
TiO_2_ with spin/scattering layers	0.0:0.0	12.70 ± 0.13
TiO_2_–Au:CNTs with spin/scattering layers	0.10:0.014	16.10 ± 0.10

The long‐term stability of the QD‐based PEC devices is an important factor to be considered for commercialization. The photocurrent density versus time curves of PEC device based QDs/TiO_2_–Au:CNTs and QDs/TiO_2_ measured at 0.6 V versus RHE under continuous 1 sun illumination (AM 1.5G, 100 mW cm^−2^) are displayed in Figure 7d. The normalization was applied by diving photocurrent density with its maximum value to visualize the decay rate. The PEC device based on QDs/TiO_2_–Au:CNTs maintaining 64% of its initial value of photocurrent density after 2 h of continuous illumination, whereas the PEC device based on QDs/TiO_2_ maintained only 50%. This enhancement in long‐term stability of the PEC device with the incorporation of Au:CNTs hybrid network in TiO_2_ mesoporous film is ascribed to several physical and mechanical phenomena occurring in the TiO_2_ mesoporous film. As discussed in previous reports,^[^ ^56^ ^]^ under 1 sun illumination, mesoporous TiO_2_ films undergo structural defects, which affects the carrier‐transport properties and hence reduces the overall long‐term stability of PEC devices. In this TiO_2_–Au:CNTs hybrid mesoporous film, CNTs partially absorb the UV light and act as blocking agent^[^ ^57^ ^]^ as well as provide better connectivity between the TiO_2_ NPs,^[^ ^58^ ^]^ whereas the presence of Au NPs also reduces carrier recombination. These promising synergetic effects of CNTs and Au NPs lead to an improved long‐term stability of QDs/TiO_2_–Au:CNT based PEC devices compared to QDs/TiO_2_.

In addition, QDs/TiO_2_–Au:CNTs hybrid photoanodes were used as photoanodes to fabricate QD sensitized solar cells (QDSCs). Figure S10a in the Supporting Information displays the *J*–*V* curves of QDSCs based on QDs/TiO_2_ and QDs/TiO_2_–Au:CNTs hybrid photoanodes under 1 sun illumination (AM 1.5G, 100 mW cm^−2^); the calculated photovoltaic parameters of QDSCs are reported in Table S3 in the Supporting Information. QDSCs based on QDs/TiO_2_–Au:CNTs hybrid photoanodes yield the PCE of 3.56%, which is 40% higher than the PEC of QDSCs based on the QDs/TiO_2_ fabricated under identical experimental condition and components (Pt counter electrode and iodide redox couple electrolyte).

We also studied the carrier dynamics at the QDs/TiO_2_–Au:CNTs/electrolyte interface by transient photovoltage decay. As shown in Figure S10b in the Supporting Information, *V*_oc_ decay is faster in the QDSC based on QDs/TiO_2_ than that of QDSC based on QDs/TiO_2_–Au:CNTs. The calculated *τ*_e_ by using Equation (1) of the respective QDSCs confirms that nonradiative carrier recombination at QDs/TiO_2_–Au:CNTs/electrolyte interface is supressed compared to QDs/TiO_2_. At a particular value of *V*_oc_ (0.3 mV), the decay rate of the calculated *τ*_e_ for QDSC based on QDs/TiO_2_ is higher than that of *τ*_e_ for QDSC based on QDs/TiO_2_–Au:CNTs, which is consistent with the obtained photovoltaic performances of the respective QDSCs (Figure S10c, Supporting Information). This suggests the versatility of TiO_2_–Au:CNTs hybrid anode for emerging solar technologies.

## 3 Conclusions and Perspectives

In conclusion, we demonstrated that the synergetic effects of plasmonic Au NPs and CNTs to boost the performance of PEC device for H_2_ generation. PEC devices based on QDs/TiO_2_–Au:CNTs hybrid photoanode with optimized amount of Au:CNTs (wt%) yields a saturated photocurrent density of 16.10 ± 0.10 mA cm^−2^ (at 1.0 V vs RHE) under standard 1 sun illumination (AM 1.5G, 100 mW cm^−2^), which is ≈26% higher than the control PEC device. Carrier dynamics studies highlight that the addition of a precise amount of Au:CNTs hybrid network in the TiO_2_ mesoporous films enhances the electron lifetime as well electron injection rate and reduces the *R*_ct_ compared to bare TiO_2_ mesoporous films. This is consistent with the obtained photocurrent density of PECs devices based on the respective photoanodes. In addition, we presented theoretical calculations supporting the role of the Au NPs in increasing the device photocurrents by injecting hot electrons into the TiO_2_–CNT hybrid matrix and, primarily, enhancing the “g‐QD” absorption rates, while showing that their size is too small to contribute as scattering centers in the photoanode.

These results provide fundamental insights about the hybrid network of QDs/Au:CNTs and offer a promising solution to improve the performance of PEC and other optoelectronic devices. Future directions will focus on the optimization of Au:CNTs hybrid networks with different size and shape of Au NPs (e.g., nanorods, nanocages, and stars) to understand the dynamics of hot‐electrons and to further improve the performance of PEC water splitting and other optoelectronic devices. The hybrid anode could be used for other optoelectronic devices, such as dye‐sensitized solar cells (DSSCs), QDSCs, photocatalytic water splitting, etc.

## 4 Experimental Section

##### Materials

Sulfur (100%), oleylamine (OLA) (technical grade, 70%), cadmium oxide (99%), cadmium nitrate tetra hydrate (≥99%), oleic acid (OA), Rhodamine 6G and octadecene (ODE), selenium pellet (≥99.999%), trioctyl phosphine oxide (TOPO), trioctyl phosphine (TOP) (97%), hexane, zinc acetate dihydrate (98%), PEI, , Na_2_S, sodium hydroxide, Na_2_SO_3_, toluene, methanol, acetone, ethanol, and isopropanol (IPA) were obtained from Sigma‐Aldrich Inc. Au (III) chloride solution 30 wt% in dilute HCl (HAuCl_4_) was purchased from Aldrich (Germany). Titania paste (code18NR‐AO) consisting of a blend of active anatase particles (20 nm in diameter) and larger anatase scattering particles (up to 450 nm in diameter) was supplied by Dyesol (Queanbeyan, Australia). FTO coated glass substrates with sheet resistance of 10 Ω square^−1^ were purchased from Pilkington glasses. All chemicals were used as received.

##### Au NPs Synthesis

Au NPs coated with bPEI were synthesized as reported previously.^[^ ^59^ ^]^ Briefly, 6.8 × 10^−2^ × 10^−3^ m HAuCl_4_ was mixed with 10 mL 25 kDa bPEI solution at a concentration of 10 mg mL^−1^. The reaction pH was adjusted to 9.5 with 0.5 m sodium hydroxide and was mixed for 2 min at room temperature. Then, the reaction mixture was placed in an oil bath at 95 °C and stirred with a mechanical stirrer at a constant speed of 350 rmp for 35 min. After cooling down the solution to room temperature, Au NPs were washed with deionized water with 30 kDa molecular weight cut‐off ultracentrifuge filter at 4500 rpm. The resulting GNP dispersions were stored in a vial covered with aluminum foil and kept at 4 °C.

##### Au:CNTs Hybrid Network Preparation

Au:CNTs hybrid network solutions with different relative ratios of Au NPs and CNTs were prepared by hydrothermal synthesis. Briefly, a homogeneous dispersion of CNTs (an average length of 10 µm) in ethanol was prepared by mixing 6 mg of CNTs in 15 mL of ethanol and sonicated for 3 h. Then, a precise amount of aqueous solution of Au NPs was mixed with ethanolic dispersion of CNTs with varying relative volume contents of Au (0.04 mg µL^−1^) and CNTs dispersion (1:1; 2:1; 3:1, and 4:1). After 1 h of ultrasonication, mixture of Au NPs and CNTs dispersion was heated to 90 °C for overnight, until the whole solvent was evaporated. Then, the precise amount of ethanol was added and again ultrasonicated for 1 h.

##### Anode Preparation

A thin and compact TiO_2_ blocking layer was deposited on ultrasonically cleaned FTO glass substrates by hydrolysis of 0.50 × 10^−3^ m TiCl_4_ solution at 70 °C for 30 min. Then, it was annealed at 500 °C for 30 min under ambient atmosphere and left to cool down to room temperature. A precise amount of ethanolic suspension of Au:CNTs was mixed into a known weight of TiO_2_ paste composed of small (20 nm in diameter) and large (up to 450 nm in diameter) size anatase particles (18NR‐AO) to prepare TiO_2_–Au:CNTs hybrid pastes. Subsequently, the above prepared TiO_2_–Au:CNTs hybrid pastes with different wt% of Au:CNTs, were deposited on top of the compact TiO_2_ layer by tape casting. A drying process was followed for 15 min at ambient conditions and then placed on a hot plate for 6 min at 120 °C. A second layer was then deposited on the top, following the same procedure. All the photoanodes were then annealed at 500 °C for 30 min under ambient conditions. For a systematic comparison, bare TiO_2_ photoanodes were also prepared under the same conditions. The thickness of all photoanodes was measured using a profilometer.

##### EPD of the QDs on the TiO_2_–Au:CNTs Hybrid Film

TiO_2_–Au:CNTs hybrid and TiO_2_ mesoporous films grown on FTO substrate were vertically immersed in the QDs dispersion in such a way that the deposited films were facing each other. The distance between them was adjusted at around 1 cm and a direct current (DC) bias of 200 V was applied for 2 h. To wash off unabsorbed QDs after the EPD process, the samples were rinsed several times with toluene and dried with N_2_. Prior to ZnS capping, the photoanodes underwent three SILAR cycles of ligand exchange of methanolic solution cetyl‐trimethyl ammonium bromide (CTAB) and toluene for 1 min dipping. After CTAB treatment, 1 min dipping in methanol was applied to wash and remove the chemical residuals from the surface and dried with N_2_. Then, 1 min dipping in toluene was applied and dried with N_2_ to complete one SILAR cycle. Then, ZnS capping layer was applied by using the SILAR. In a typical SILAR deposition cycle, first Zn^2+^ ions were deposited from a methanolic 0.1 m solution of Zn(OAc)_2_. Then, sulfide precursor was 0.1 m solution of Na_2_S in the mixture of methanol and water. One complete SILAR cycle consisted of 1 min of dip‐coating the TiO_2_ photoanode into the Zn^2+^ precursors, and subsequently into the S^2−^ solutions. After each bath, to remove the unabsorbed chemical residuals, the photoanode was thoroughly rinsed with corresponding solvent (methanol or mixed solution), respectively, and dried with N_2_. There were two SILAR cycles applied to form the ZnS capping layer.

##### Characterizations

Small angle XRD of extensively purified QDs was carried out with a Philips X′pert diffractometer using a Cu K_*α*_ radiation source (*λ* = 0.15 418 nm). TEM and HR‐TEM images of Au, Au:CNTs, and TiO_2_–Au:CNTs film were collected by using a JEOL 2100F TEM system. Field emission scanning electron microscopy (FE‐SEM, JEOL JSM‐6900 F) was applied to study the morphology of the TiO_2_ and TiO_2_–Au:CNTs hybrid photoanodes. The chemical composition mapping of QD sensitized TiO_2_ photoanodes was carried out by EDS. The Raman spectra of the photoanode were measured with a 514 nm excitation source. The UV–vis absorption spectra were recorded with a Cary 5000 UV‐Vis‐NIR spectrophotometer (Varian) with a scan speed of 600 nm min^−1^. Fluorescence spectra were taken with a Fluorolog‐3 system (Horiba Jobin Yvon). The PL lifetime of the QDs was measured in the time‐correlated single‐photon counting (TCSPC) mode with a 444 nm laser. XPS was performed in a VG Escalab 220i‐XL equipped with hemispherical analyzer, applying a Twin Anode X‐Ray Source. UPS measurement was performed on a VG ESCALAB 3 Mark II high vacuum system.

The PEC performance of the photoanodes was evaluated in a typical three‐electrode configuration, consisting of a QDs/TiO_2_–Au:CNTs photoanode as a working electrode, a Pt counter electrode, and a saturated Ag/AgCl reference electrode. An insulating epoxy resin was used to cover the sample's surface except for the active area, to avoid any direct contact between the electrolyte and the conducting back‐contact and/or the connecting wire. Subsequently, the sample was fully immersed in the electrolyte containing 0.25 m Na_2_S and 0.35 m Na_2_SO_3_ (pH ≈13) as the sacrificial hole scavenger. All potentials, measured with respect to the reference electrode of Ag/AgCl during the PEC measurements, were converted to scale according to the following equation *V*_RHE_ = *V*_Ag/AgCl_ + 0.197+ pH × (0.059). The photoresponse was measured by using a 150 W Xenon lamp as a light source with an AM 1.5G filter. The sample was placed at a distance of 2 cm from the lamp case (7 cm far from the actual bulb). Prior to each measurement, light intensity was monitored by a thermopile and adjusted to 100 mW cm^−2^. All the current versus potential measurements were carried out at a 20 mV s^−1^ sweep rate.

Electrochemical impedance spectroscopy (EIS) was carried out under dark conditions by using a SOLARTRON 1260 A Impedance/Gain‐Phase Analyzer with a bias voltage from 550 to 750 mV. The tests were recorded over a frequency range between 10 mHz and 300 kHz, and the AC signal was 10 mV in amplitude. All impedance measurements were analyzed using an appropriate equivalent circuit model with Z‐View software (v3.5, Scribner Associate, Inc.).

## Conflict of Interest

The authors declare no conflict of interest.

## Supporting information

Supporting Information
