# Supplementary material for: Synergistic Effect of Plasmonic Gold Nanoparticles Decorated Carbon Nanotubes in Quantum Dots/TiO2 for Optoelectronic Devices
Source: Adv Sci (Weinh). 2020 Aug 26;7(20):2001864. doi: 10.1002/advs.202001864 (PMC7578890; doi:10.1002/advs.202001864)
Supplement: Supplementary file 1 — Supporting Information [file ADVS-7-2001864-s001.pdf]

© 2020 Wiley-VCH GmbH

ADVANCED SCIENCE
Open Access

Supporting Information

for *Adv. Sci.,* DOI: 10.1002/advs.202001864

Synergistic Effect of Plasmonic Gold Nanoparticles Decorated Carbon Nanotubes in Quantum Dots/TiO_2 for Optoelectronic Devices

Gurpreet Singh Selopal, Mahyar Mohammadnezhad, Lucas V. Besteiro, Ozge Cavuslar, Jiabin Liu, Hui Zhang, Fabiola Navarro-Pardo, Guiju Liu, Maorong Wang, Emek G. Durmusoglu, Havva Yagci Acar, Shuhui Sun, Haiguang Zhao, Zhiming M. Wang,* and Federico Rosei**

Supporting information

Synergistic Effect of Plasmonic Gold Nanoparticles Decorated Carbon Nanotubes in Quantum Dots/TiO_2 for Optoelectronic Devices

Gurpreet Singh Selopal, Mahyar Mohammadnezhad, Lucas V. Besteiro, Ozge Cavuslar, Jiabin Liu, Hui Zhang, Fabiola Navarro-Pardo, Guiju Liu, Maorong Wang, Emek G. Durmusoglu, Havva Yagci Acar, Shuhui Sun, Haiguang Zhao, Zhiming M. Wang* and Federico Rosei**

Dr. G. S. Selopal, Dr. L.V. Besteiro, Dr. F. Navarro-Pardo, Prof. Z. M. Wang
Institute of Fundamental and Frontier Sciences
University of Electronic Science and Technology of China
Chengdu 610054, P. R. China.
E-mail: zhmwang@uestc.edu.cn

Dr. G. S. Selopal, Dr. M. Mohammadnezhad, Dr. L.V. Besteiro, J. Liu, H. Zhang, Dr. F. Navarro-Pardo, Prof. S. Sun, Prof. F. Rosei
Centre Énergie, Matériaux et Télécommunications, Institut National de la Recherché Scientifique, 1650 Boul. Lionel Boulet, J3X 1S2 Varennes, Québec, Canada
Email: rosei@emt.inrs.ca

Dr. O. Cavuslar, Dr. E. G. Durmusoglu, Prof. H. Y. Acar
Department of Chemistry, Koc University, Rumelifeneri Yolu, Sariyer, Istanbul Turkey

G. Liu, M. Dr. Wang, Prof. H. Zhao
State Key Laboratory of Bio-Fibers and Eco-Textiles & College of Physics, Qingdao University, No. 308 Ningxia Road, Qingdao 266071, PR China.
Email: hgzhao@qdu.edu.cn

Synthesis of CdSe QDs and core/shell QDs

CdSe QDs were synthesized using the hot-injection approach [1]. The deposition of CdS layers on CdSe QDs followed procedures described elsewhere [2]. Typically, in a 50-mL round-bottom flask, OLA (5 ml), ODE (5 mL) and CdSe QDs (~2×10^{-7} mol in hexane) were degassed at 110 °C for 30 min. Then N_2 was restored into the reaction flask and the reaction temperature was raised to 240 °C with continuous stirring. The $Cd(OA)_2$ dissolved

in ODE (0.25 mL, 0.2 M) was injected dropwise and wait for 2.5 h to complete the reaction, followed by a dropwise injection of 0.2 M sulfur dissolved in ODE with the same volume. The shell was further annealed for 1 h. All shells were annealed at 240 °C for ~10 min following the addition of sulfur and ~2.5 h after the dropwise injection of the $Cd(OA)_2$ in ODE. Graded alloyed shells were grown over the CdSe core QDs by tailoring the molar ratio of S:Se during in situ growth of each layer $CdSe_xS_{1-x}$ (x = 0.9~0.1)[3]. Subsequently, another one of CdS was coated on the alloyed shell. The reaction was cooled to room temperature using ice-cold water. Then ethanol was added, and the suspension was centrifuged. The supernatant was removed. Finally, the QDs were then dispersed in toluene for further characterization.

Simulation of optical properties of Au NPs

As the AuNPs are integrated in the annealed mixture with TiO_2 and MWCNT, we can consider that they are experiencing an effective dielectric medium which is fundamentally that of TiO_2. However, it is expected that the final aggregate will present small porous irregularities in accommodating the multiple nanostructures. Therefore, we have calculated the effective medium's permittivity ε_{m} assuming a total of 5% volume of empty space, i.e. $f_{\mathrm{i}} = 0.05$, in the annealed aggregate, and using the Maxwell-Garnett equation for spherical incrustations with permittivity $\varepsilon_{\mathrm{i}} = 1.33^2$ in a continuous medium with permittivity $\varepsilon_{\mathrm{h}} = 2.55^2$

$$\varepsilon_{\mathrm{m}} = \varepsilon_{\mathrm{h}} \frac{2\varepsilon_{\mathrm{h}} + \varepsilon_{\mathrm{i}} + 2 f_{\mathrm{i}} \;\; \varepsilon_{\mathrm{i}} - \varepsilon_{\mathrm{h}}}{2\varepsilon_{\mathrm{h}} + \varepsilon_{\mathrm{i}} - f_{\mathrm{i}} \;\; \varepsilon_{\mathrm{i}} - \varepsilon_{\mathrm{h}}}$$

We utilize this value for the dielectric index of the medium in the numerical electrodynamic calculations of the system, together with experimental permittivity values for gold. [4] These simulations were conducted using finite element methods (FEM), using the commercial software

package COMSOL, and modeled an isolated AuNP immersed in a homogeneous effective dielectric medium.

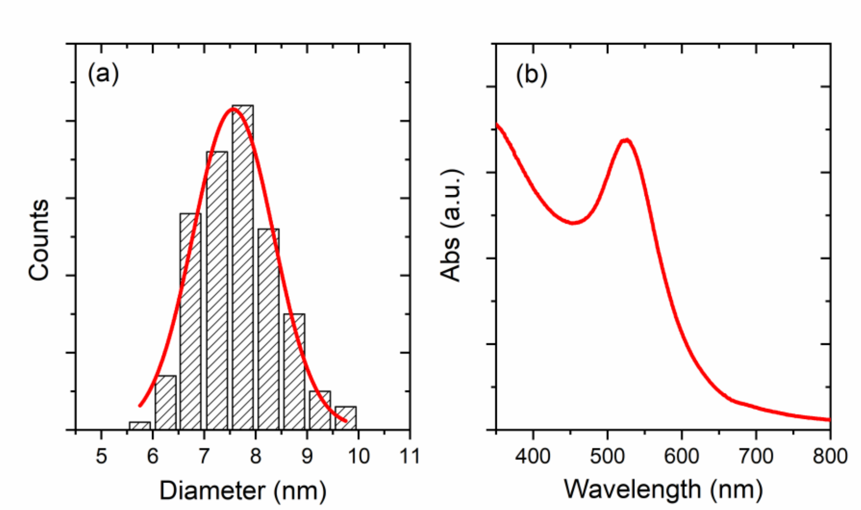

Figure S1. PEI coated as synthesized Au nanoparticles: (a) Size distribution; (b) Absorption.

Table S1. Raman measurement parameters of CNTs and Au:CNTs hybrid network

Sample	G-band (λ_G) (nm)	D-band (λ_D) (nm)	G-band intensity (I_G) (a.u.)	D-band intensity (I_D) (a.u.)	I_D/I_G
**CNTs**	1578.02	1345.65	28472.45	34902.04	1.23
**Au:CNTs (0.10 : 0.014)**	1582.57	1346.33	23022.90	22661.90	0.98

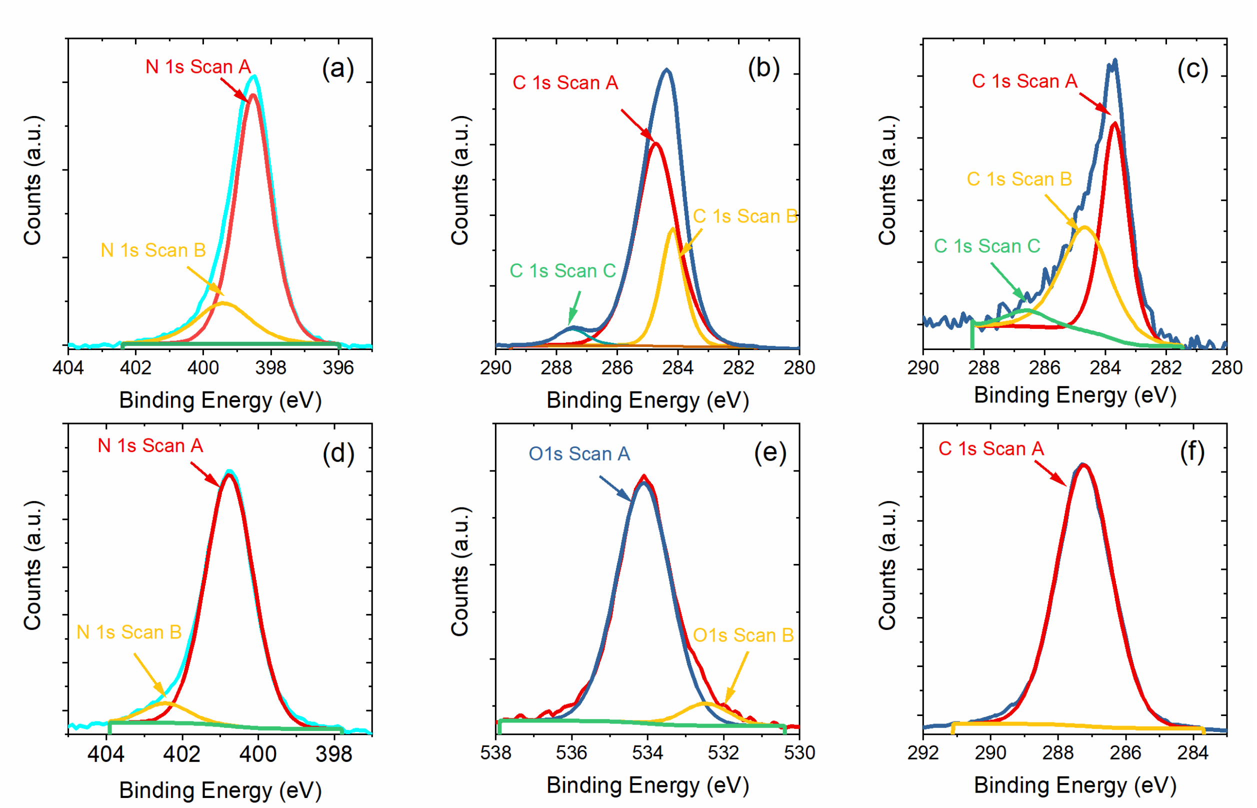

Figure S2. XPS of: Au:CNTs hybrid network: (a) N 1s; (b) C 1s; CNTs: (c) C 1s and Au NPs: (d) N 1s; (e) O 1s; (f) C 1s.

Table S2. XPS analysis of Au NPs and Au:CNTs hybrid network

Sample	B.E. (eV)	Atomic (%)
**Au-PEI**		
N 1S A	400.06	28.94
N 1S B	401.74	2.18
**Au:CNTs**		
N 1S A	400.43	21.96
N 1S B	401.97	4.45

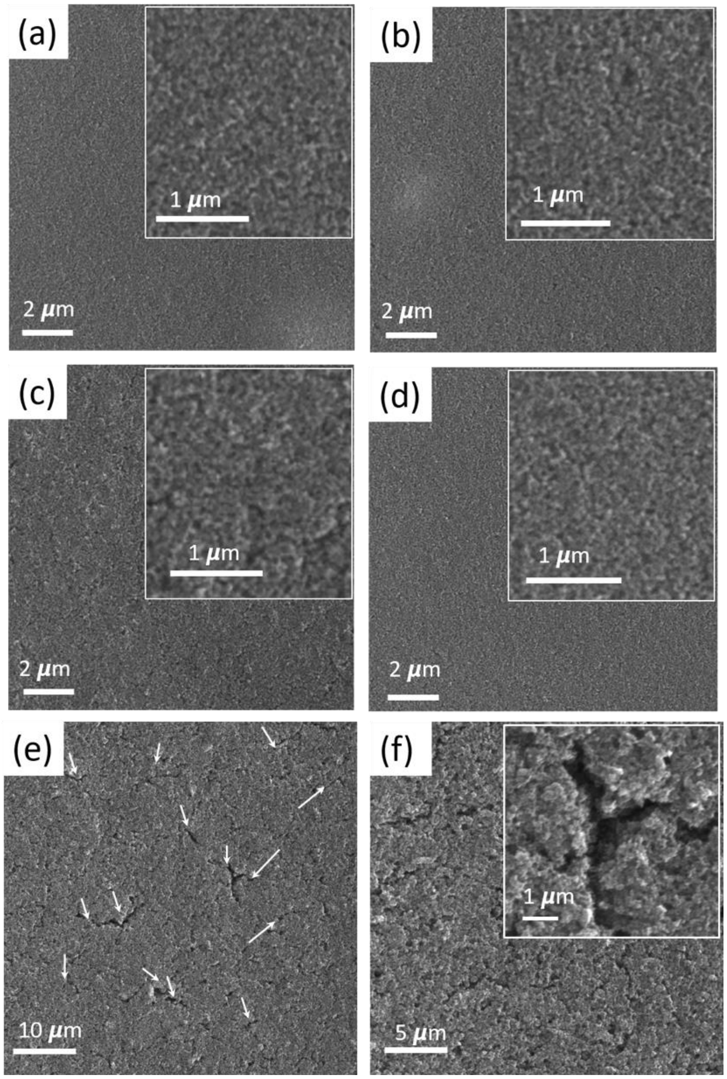

Figure S3. SEM images of: (a) TiO_2; (b) TiO_2-Au; (c) TiO_2-CNTs; (d) TiO_2-Au: CNTs (0.10: 0.014 wt %); and (e)-(f) TiO_2-Au: CNTs (0.15: 0.014 wt %) hybrid mesoporous anodes. The inset of a, b, c, d and f displays the high-resolution SEM images of corresponding hybrid film.

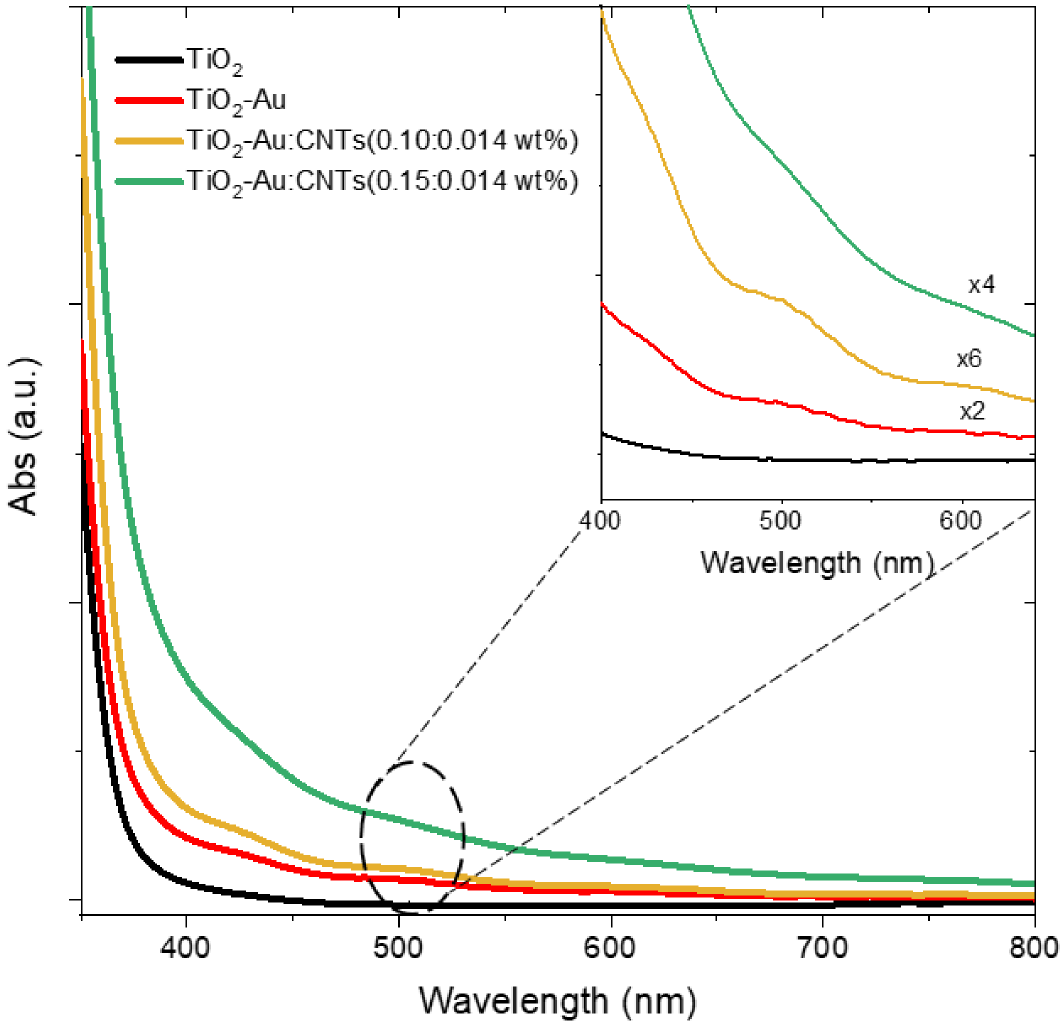

Figure S4. Absorption spectra of TiO_2 and TiO_2-Au:CNTs photoanodes at different content of Au:CNTs hybrid network

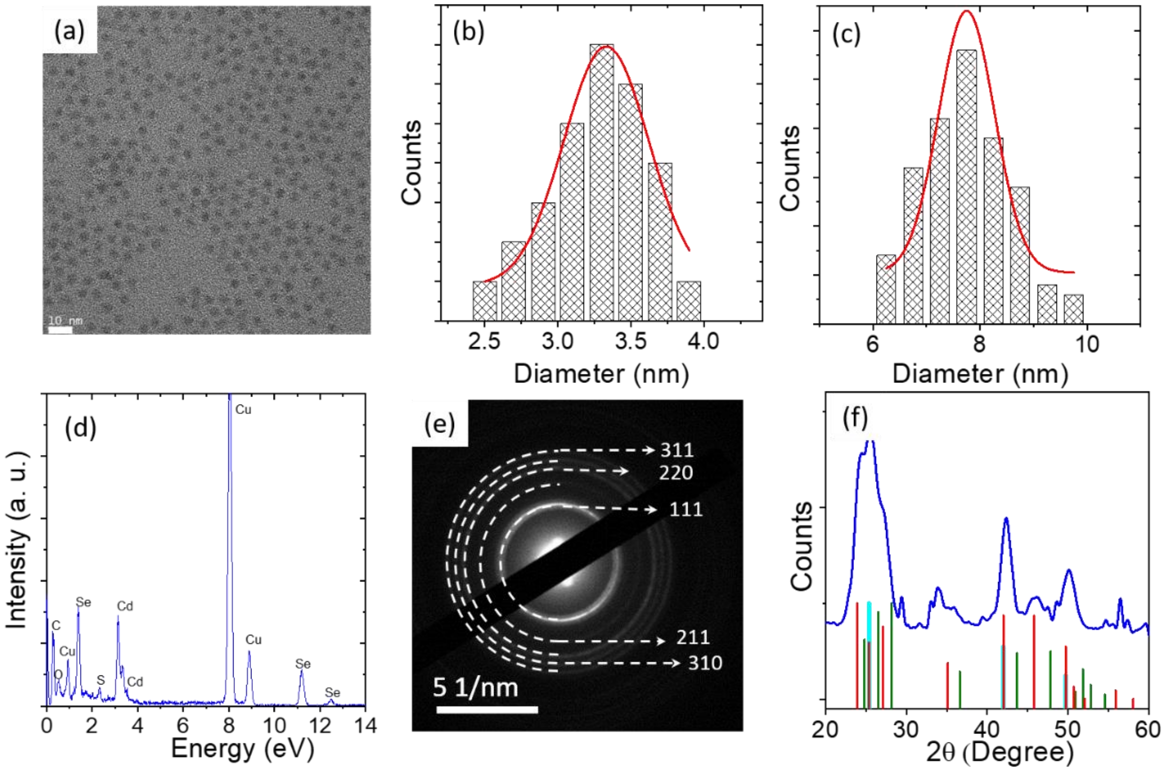

Figure S5. CdSe core QDs: (a) TEM; (b) size distribution. "g-QDs": (c) size distribution (d) EDS; (e) SEAD; (f) XRD pattern. The Joint Committee on Powder Diffraction Standards (JCPDS) card files for CdSe (00190191, dark green dashed for ZB and 08-459, cyan for WZ) and CdS (01-077-2306, red for WZ) are displayed for phase identification.

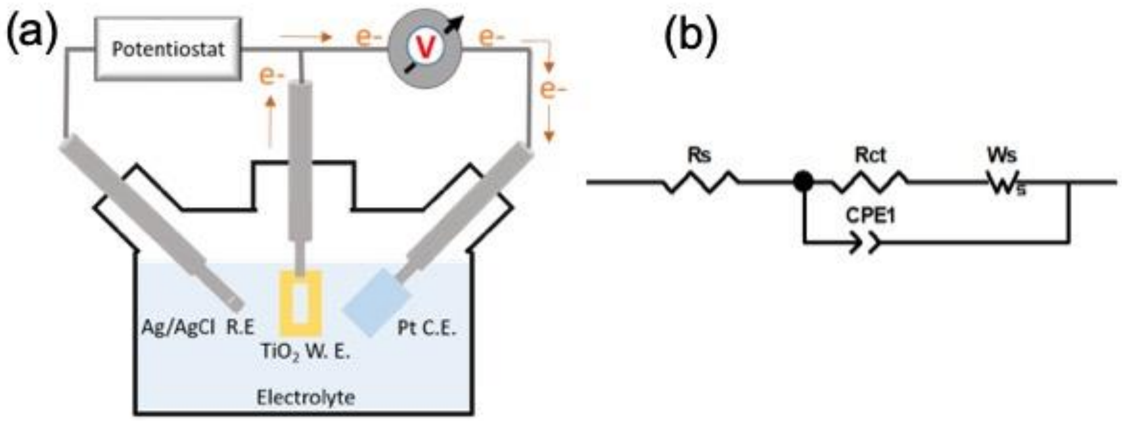

Figure S6. (a) Schematic of three-electrodes configuration for EIS measurements; (b) An equivalent circuit used to fit the mid-frequency range data.

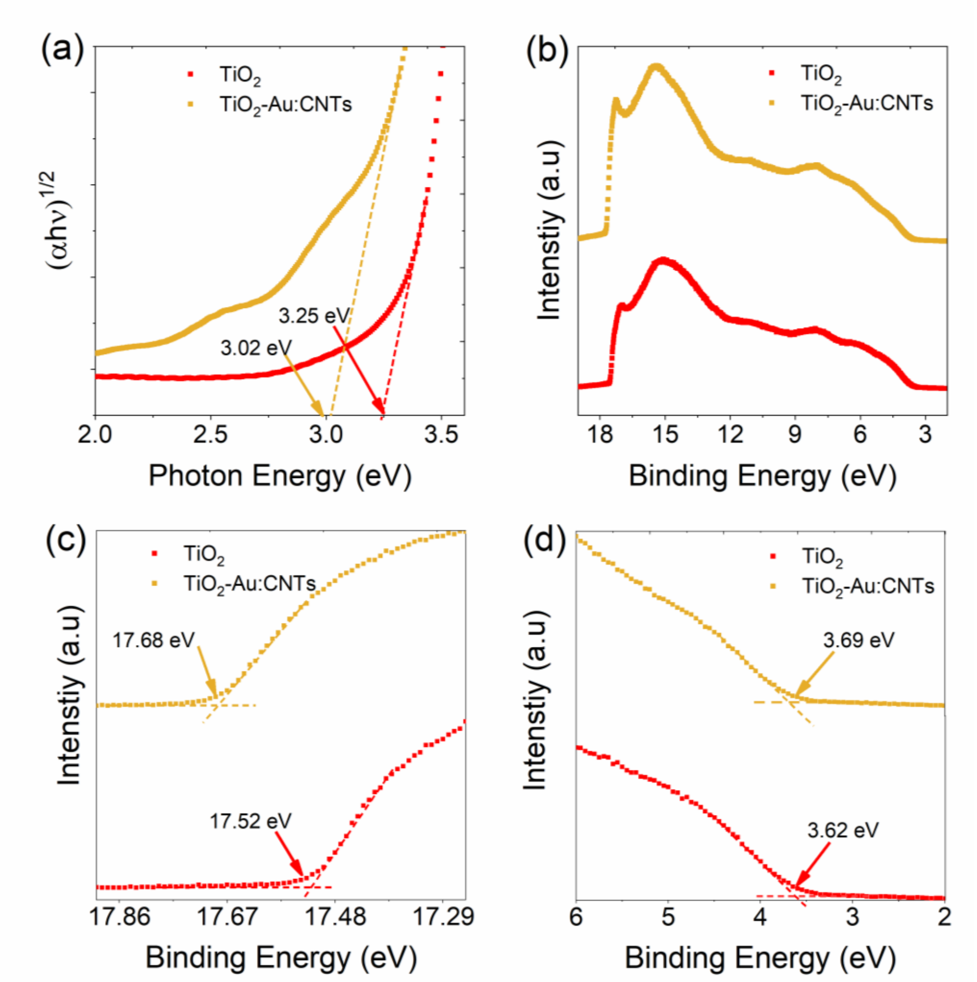

Figure S7. Electronic band alignment of TiO_2 and TiO_2-Au:CNTs hybrid photoanode: (a) Tauc plots; (b) UPS spectra; (c) High binding energy cut-off for Fermi level estimation; (d) Low-binding energy cut-off for the valence band maximum (VBM).

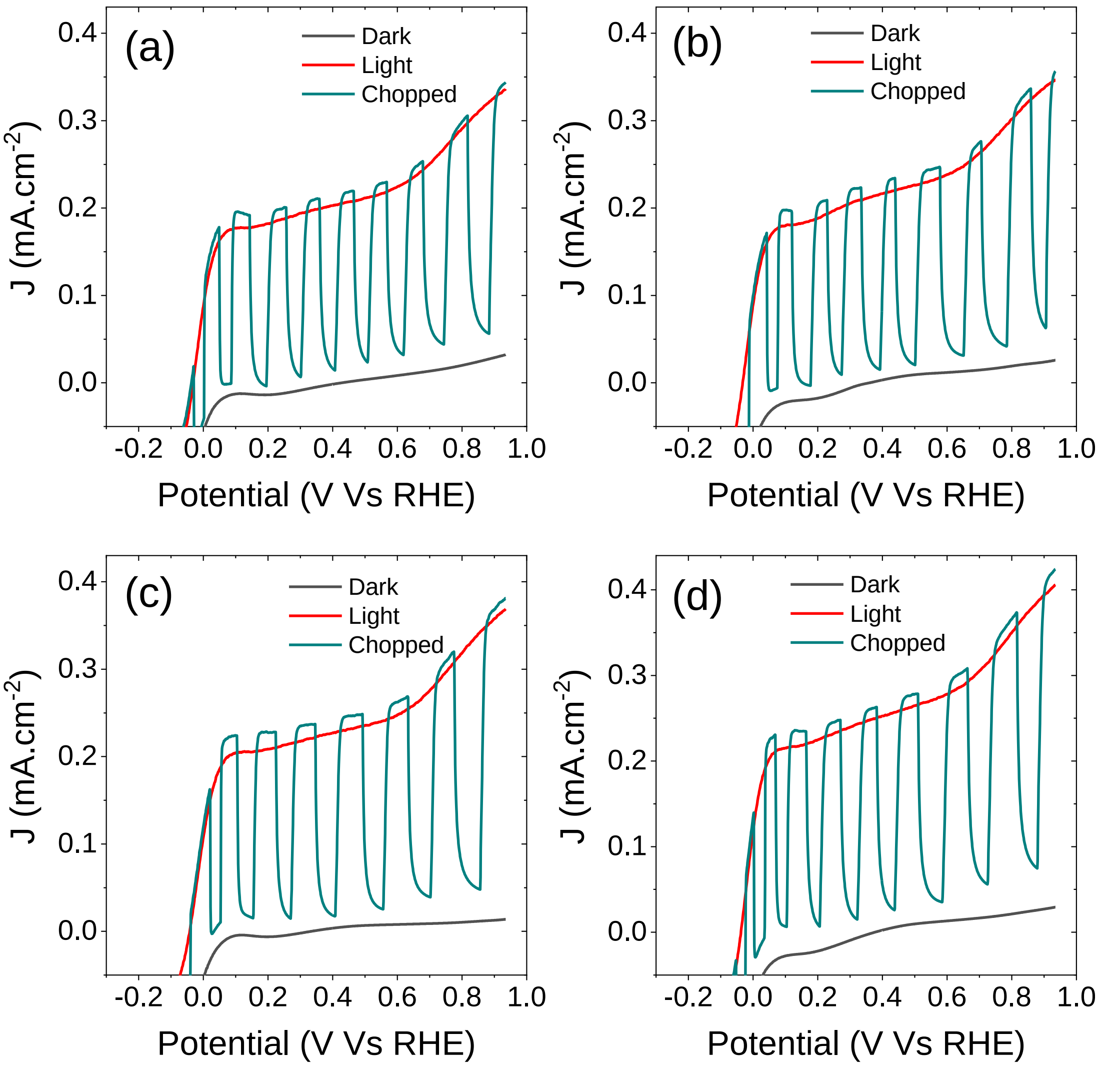

Figure S8. Photocurrent density vs potential (vs RHE) of PEC devices under dark, chopped and continuous one sun light illumination (AM 1.5G, 100 mW·cm^{-2}): (a) TiO_2; (b) TiO_2-CNTs; (c) TiO_2-Au; (d) TiO_2-Au:CNTs

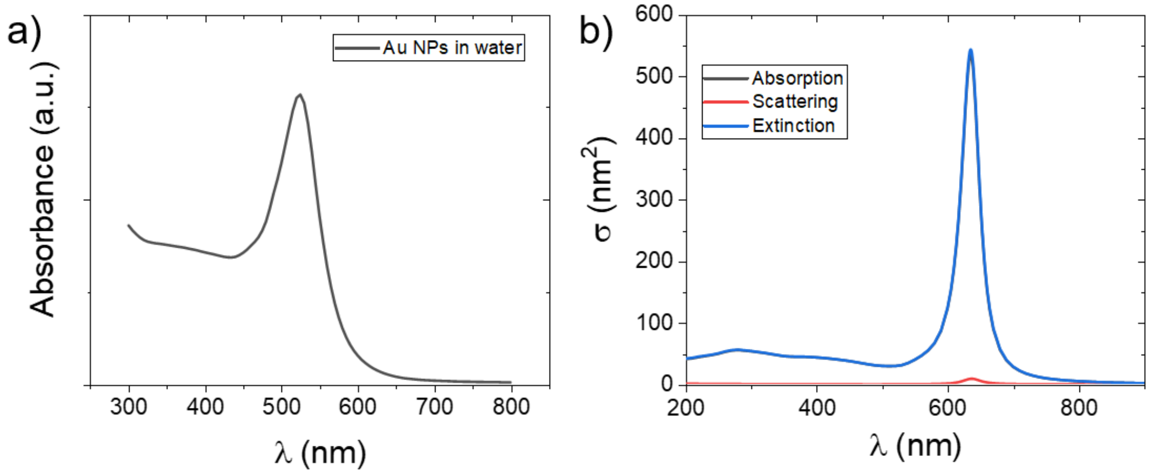

Figure S9. Theoretical results. (a) Absorbance of 10 nm Au NPs in water solution. (b) Cross sections for 10 Au NPs immersed in an effective medium with a dielectric index of 2.5, as estimated through the method described above. As we can see, the expected scattering arising from such small Au NPs is negligible.

QDSCs measurements:

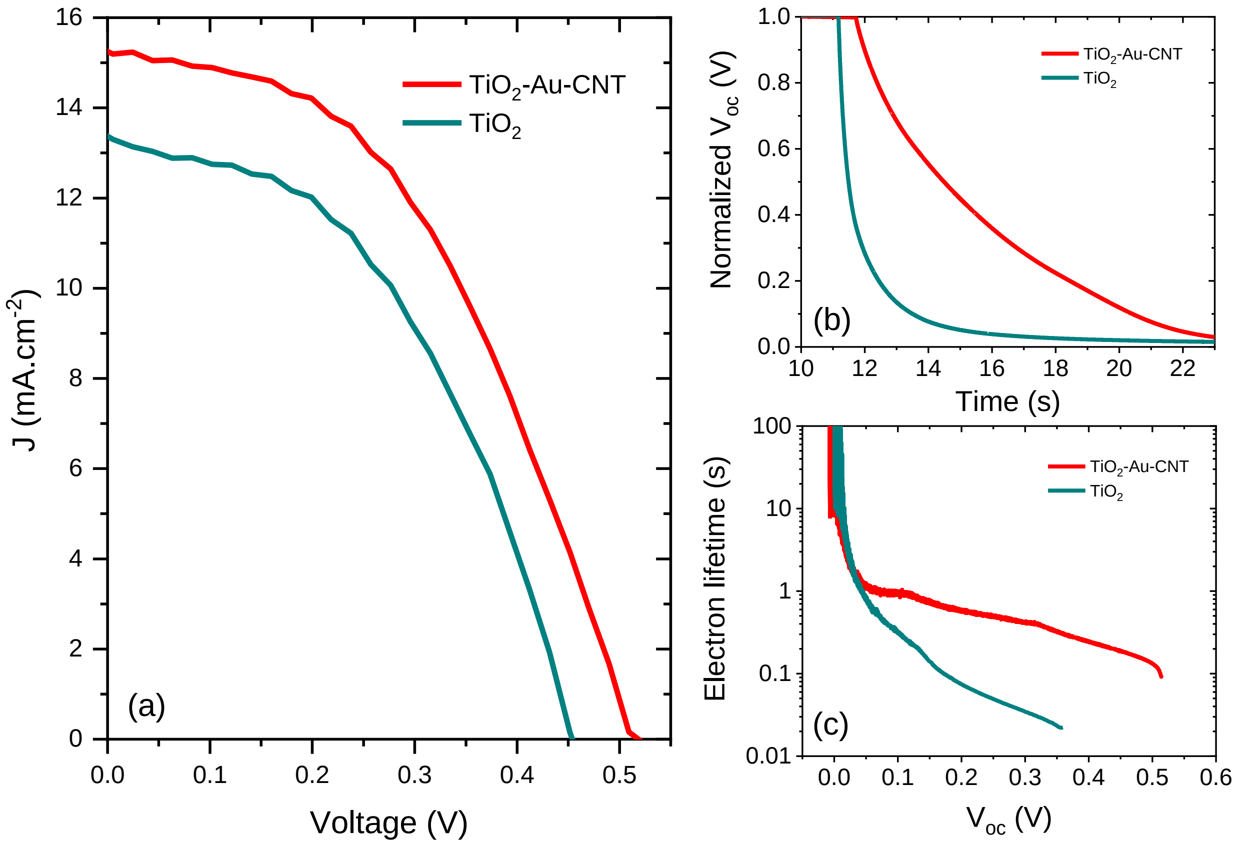

Figure S10. (a) Current density vs voltage curves of QDSCs under one sun illumination based on different types of Photoanodes: dark grey based on TiO_2 and red line based TiO_2-Au:CNTs (0.10:0.014 wt%). Transient photovoltage decay measurements of corresponding QDSCs: (b) Normalized V_{oc} versus time (s); (c) calculated electron lifetime (τ (s)) versus V_{oc}.

Table S3. Calculated photovoltaic parameters of QDSCs under one sun illumination (AM 1.5 G, 100 mW·cm^{-2}) based on QDs/ TiO_2-Au:CNTs and QDs/TiO_2 photoanodes reported in **Figure S9**.

**Anode structure**	**J_{sc} (mA.cm^{-2})**	**V_{oc} (V)**	**FF (%)**	**PCE (%)**
TiO_2	13.37	0.454	46	2.78
TiO_2-Au:CNTs	15.24	0.518	45	3.56

References

[1] B.O. Dabbousi, J. Rodriguez-Viejo, F.V. Mikulec, J.R. Heine, H. Mattoussi, R. Ober, K.F. Jensen and M.G. Bawendi, *J. Phys. Chem. B,* **1997**, *101*, 9463.

[2] Y. Ghosh, B. D. Mangum, J. L. Casson, D. J. Williams and H. Htoon, J. A. Hollingsworth, *J. Am. Chem. Soc*, **2012**, *134*, 9634.

[3] G. S. Selopal, H. G. Zhao, G. J. Liu, H. Zhang, X. Tong, K. H. Wang, J. Tang, X. H. Sun, S. H. Sun, F. Vidal, Y. Q. Wang, Z. M. M. Wang, F. Rosei, *Nano Energy*, **2019**, *55*, 377.

[4] Johnson, P. B.; Christy, R. W., Optical Constants of Noble Metals. *Phys Rev B* **1972,** *6*, 4370.
